# Integrating decision trees with ANOVA for enhanced interaction visualisations

**DOI:** 10.1371/journal.pone.0357663

**Published:** 2026-09-08

**Authors:** Perseverence Savieri, Lara Stas, Kurt Barbé

**Affiliations:** 1 Research Center for Digital Medicine (REDM), Vrije Universiteit Brussel (VUB), Brussels, Belgium; 2 Core Facility - Support for Quantitative and Qualitative Research (SQUARE), Vrije Universiteit Brussel (VUB), Brussels, Belgium; University of Lahore - Raiwind Road Campus: The University of Lahore, PAKISTAN

## Abstract

Interaction effects in ANOVA provide crucial insights into how the effect of one independent variable depends on the level of another. However, interpreting these interactions, especially three-way interactions, remains a challenge. Firstly, visualisation techniques typically rely on multiple separate two-dimensional plots conditioned on the levels of a third factor; as the number of levels of the third factor increases, so does the number of plots, complicating interpretation. Secondly, routinely applying uncorrected post-hoc pairwise comparisons oversimplifies interaction effects and, by ignoring the multiplicity of tests, inflates Type I error rates, potentially leading to misleading conclusions. Advanced methods, such as partial dependence plots and interaction decomposition models, have partially addressed these limitations; however, they typically require substantial statistical or computational expertise, which limits their accessibility. To address these challenges, this manuscript introduces **EXCITE** (Enhanced eXploration of Complex Interactions in ANOVA using Trees), an interactive web application developed using the Shiny package within the R statistical environment. EXCITE integrates traditional two- and three-way ANOVA models with decision tree-based visualisations. Decision trees detect interaction patterns via recursive data partitioning and present conditional subgroup relationships through an interpretable tree structure. The advantages of EXCITE include presenting two- and three-way interactions intuitively, enhancing accessibility, and facilitating accurate interpretation of complex statistical interactions. This integration provides researchers and educators across various scientific disciplines with a user-friendly tool for interpreting and visualising ANOVA interactions. We illustrate the capabilities of EXCITE using both simulated examples and a real dataset to demonstrate its practical applicability. The web application is freely available online, requiring no installation or coding expertise, at https://zq9mvv-vub0square.shinyapps.io/EXCITE-research-tool/.

## 1. Introduction

### 1.1. Background and problem statement

Analysis of variance (ANOVA) is widely used to assess mean differences across groups defined by categorical factors [1–3]. Originally developed by Sir Ronald Fisher, ANOVA’s simplicity and interpretability have led to its widespread adoption across disciplines such as psychology, neuroscience, and medicine [4]. In two- and three-way ANOVA, researchers may test not only main effects but also interactions; whether the effect of one factor depends on the level of another [5,6]. Interactions, typically visualised as non-parallel means patterns across factor combinations [7,8], are critical for understanding heterogeneity among multiple factors [1,6].

Interpreting higher-order interactions, however, remains challenging due to the limitations of traditional visualisation methods. These effects are often shown using bivariate plots, which condition on the levels of a third factor, resulting in multiple two-dimensional plots across separate panels [5,7,9]. As the number of factors or the number of levels within the conditioned factor increases, this “split-plot” approach produces an expanding series of separate panels, each displaying only a fragment of the full interaction structure [5, 10]. For a three-way interaction, this already requires examining several conditioned plots; for higher-order interactions, the number of required panels increases rapidly, making it harder to see how the factors jointly influence the outcome. As the visualisation becomes more fragmented, important patterns may be less apparent.

Researchers often turn to post hoc pairwise comparisons because omnibus ANOVA tests merely indicate whether an interaction exists, without specifying where the differences lie. These follow-up tests aim to identify which factor-level combinations differ from one another. However, as highlighted in recent reviews [4], the routine use of uncorrected post hoc comparisons can oversimplify inherently multivariate interaction patterns by reducing them to isolated mean contrasts [11,12]. This practice increases the risk of Type I error due to multiple testing and, crucially, does not resolve the core interpretability problem: it still does not provide an integrated view of how factors jointly shape the response [6,8]. Furthermore, standard ANOVA coding constraints (e.g., sum-to-zero) affect the interpretability of higher-order terms, as parameter estimates depend on the chosen contrast scheme and the parameterisation of the model [7].

### 1.2. Existing methodology

Several approaches have sought to improve the visualisation and interpretation of interaction effects [4,13]. Common methods, such as estimated marginal means plots and simple slope analyses, provide clearer insight than post hoc comparisons [5]. More advanced, machine-learning-inspired, including partial dependence plots and interaction decomposition models, capture complex relationships through graphical frameworks like ggplot2 and lattice [5,13]. However, these approaches often require substantial statistical or computational expertise, limiting accessibility for non-specialists.

Interactive tools have recently emerged to address limitations in traditional ANOVA approaches. For instance, Jiang et al. [14] developed *moreThanANOVA*, to automate distribution and variance checks with balanced post hoc testing, while Sisso et al. [15] introduced an educational web application for teaching one-way ANOVA. Yet, none of these tools explicitly target the visualisation of complex two- or three-way interactions. They focus on assumption testing or instructional contexts rather than integrated visual exploration, leaving the challenge of multi-factor interaction interpretation largely unresolved.

### 1.3. Objectives

Despite these advances in visualisation methods, significant gaps persist. Existing tools typically focus exclusively on ANOVA or machine learning visualisations [13–16], requiring users to interpret multiple-panel plots or demanding advanced statistical expertise, limiting their practical utility for non-specialists. Addressing these limitations requires a single-view visualisation that simplifies the interpretation of complex three-way interactions.

To meet this need, we introduce **EXCITE** (Enhanced eXploration of Complex Interactions in ANOVA using Trees), an interactive Shiny application that integrates traditional ANOVA with decision-tree visualisation [17]. EXCITE demonstrates how decision trees complement classical ANOVA, improving interpretability and accessibility when exploring multifactor interactions. The novelty of the proposed approach lies precisely in integrating traditional two- and three-way ANOVA with the tree-based visual exploration. Through this innovative merging of ANOVA with decision tree visualisations, **EXCITE** aims to offer researchers an accessible, interactive tool that not only simplifies but also enhances the interpretability of intricate statistical interactions, thus providing clear value and broad applicability across diverse research domains.

Importantly, EXCITE is positioned as a complement to factorial ANOVA, optimised for categorical predictors and designed to display two- and three-way interactions in a single tree-based visualisation of conditional relationships, rather than as a replacement for tools focused on assumption checking (e.g., moreThanANOVA) or model-agnostic machine-learning visualisations (e.g., partial dependence or interaction decomposition); consequently, the evaluation in this manuscript emphasises interpretability and workflow coherence with ANOVA rather than predictive benchmarking.

The Methods section outlines the theoretical framework and app design; readers interested primarily in practical use may refer directly to Section 2.4 (Workflow and User Interface Overview), where the app’s operation is demonstrated step-by-step. The Results section presents applied examples, followed by a Discussion on implications and future extensions, and a Conclusion summarising key contributions.

## 2. Methods

### 2.1. Overview of two- and three-way ANOVA

ANOVA can be conducted using either a single factor (one-way ANOVA) or multiple factors simultaneously (factorial ANOVA). When two factors are considered simultaneously, this is known as two-way ANOVA. Two-way ANOVA can assess the main effects of each factor and the potential interaction effects between these factors [3].

In a two-way factorial ANOVA design with interactions, the general linear model is expressed as:


$$\begin{array}{c} Y_{ijk}=\mu +A_{i}+B_{j}+(AB{)}_{ij}+\;\epsilon_{ijk} \end{array}$$

$Y_{ijk}$is the response for the k^th^ observation within the combination of the *i*^*th*^ level of factor A and the *j*^*th*^ level of factor B. μ denotes the overall mean response across all factor combinations. $A_{i}$ captures the main effect of the *i*^*th*^ level of factor A, $B_{j}$ captures the main effect of the *j*^*th*^ level of factor B, $(AB{)}_{ij}\;$represents the interaction effect between factors A and B, describing how the effect of factor A depends on the level of factor B, and vice versa. $\epsilon_{ijk}$ represents the error term, assumed to be independently and identically normally distributed with mean zero and constant variance $\sigma^{\text{2}}$ [1,3].

The model expands similarly for a three-way factorial ANOVA, capturing additional interactions:


$$\begin{array}{c} Y_{ijkl}=\mu +A_{i}+B_{j}+{C_{k}+(AB)}_{ij}{+\;(AC)}_{ik}\;{+\;(BC)}_{jk}\;{{+\;(ABC)}_{ijk}+\epsilon }_{ijkl} \end{array}$$

where each term incorporates an additional factor (factor C) and relevant interactions [2]. Specifically, interpreting a three-way interaction $(ABC{)}_{ijk}$ involves examining how the interaction between two factors (e.g., A and B) may vary depending on the level of a third factor (C). For instance, the interaction effect between factors A and B could change across different levels of factor C, meaning the relationship between A and B is not consistent but conditional on C. Similarly, the interaction between factors A and C may differ depending on the levels of factor B, and the interaction between factors B and C may shift according to the levels of factor A [3]. Thus, interpreting a three-way interaction involves understanding and visualising how these conditional relationships among factors differ across their combined levels.

In practice, researchers frequently omit higher-order interactions (especially beyond three-way) from models to simplify interpretation and maintain sufficient statistical power, particularly when sample sizes are limited [18–20]. However, careful model specification should always be guided by theoretical considerations and study design rather than convenience alone. This is because in some contexts, such interactions may carry meaningful insights, for example, when a treatment’s effectiveness differs across multiple demographic and contextual variables simultaneously (e.g., Therapy*Sex*Age*Setting). To balance interpretability and practical significance, this manuscript specifically focuses on two- and three-way ANOVA to clearly illustrate the integration and interpretation advantages of combining ANOVA with decision-tree visualisations.

### 2.2. Decision tree modelling

**Fig 1:**
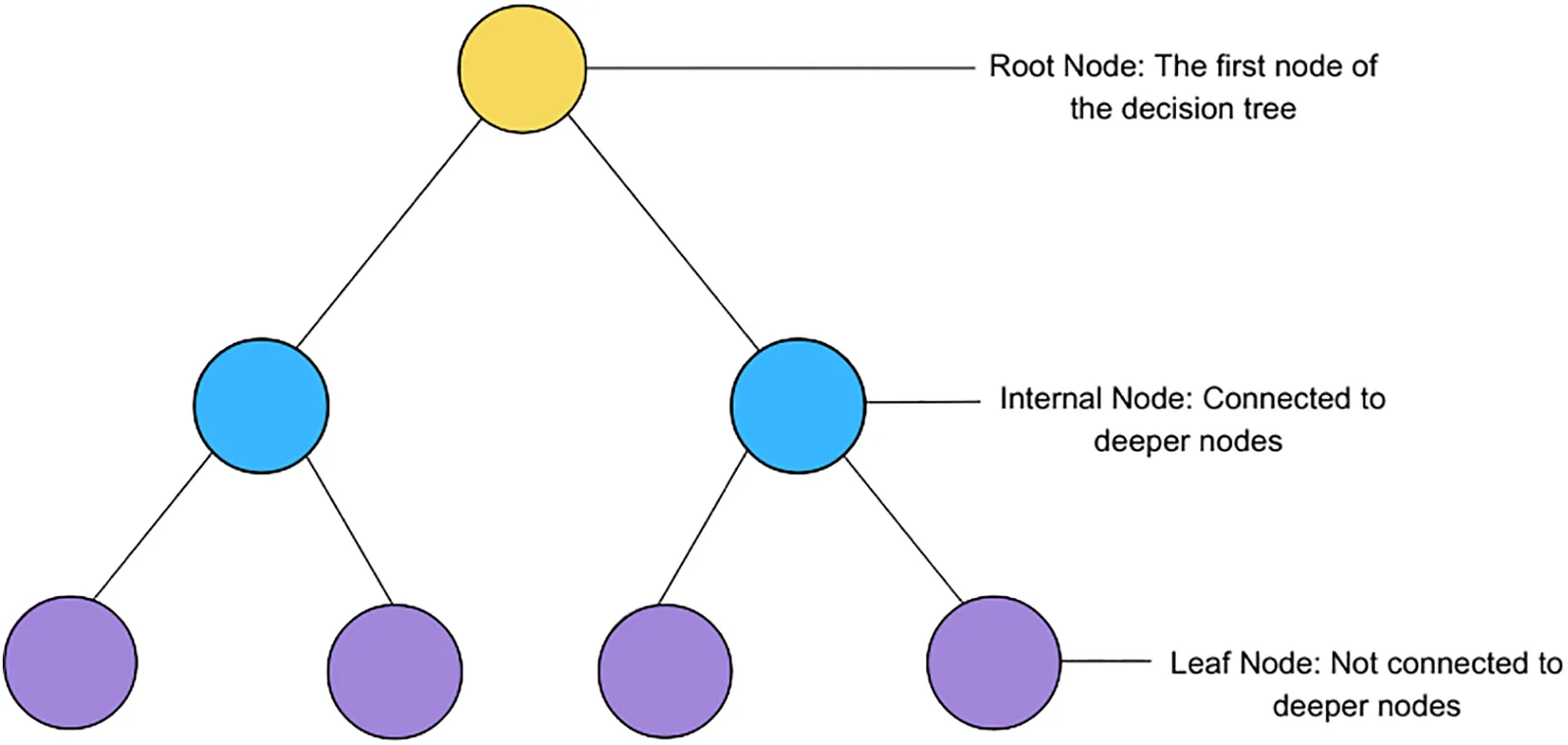
Example of a decision tree displaying hierarchical splits. Each path illustrates a unique interaction among predictor levels.

Decision trees are supervised machine learning models used for classification and regression due to their simplicity and interpretability [21,22]. They partition data hierarchically by applying recursive splits on predictor variables to form a tree-like structure composed of nodes and branches (see Fig 1). Each internal node represents a decision rule, splitting data into subsets based on a specific predictor, and each leaf node provides the final classification or prediction outcome [23,24]. Due to the transparent “if-then” logic, decision trees illustrate how different variables interact and influence outcomes. This visualisation makes complex dependencies immediately interpretable, especially for non-statistical audiences [22,25].

In the context of EXCITE, decision trees complement ANOVA by visually representing interactions detected through omnibus tests [26]. Whereas ANOVA identifies interactions statistically, decision trees reveal how factor-level combinations shape the response through recursive binary splits. Importantly, this hierarchy reflects the structure of the visual representation rather than the statistical structure of the ANOVA interaction itself. EXCITE implements the Classification and Regression Trees (CART) algorithm [27] via the *rpart* R package [28], selecting splits at each node by maximising the reduction in within-node variance. The algorithm evaluates all possible predictors and split points, choosing the partition that minimises the sum of squared errors within the resulting subsets [26]. This recursive partitioning continues until predefined stopping criteria (e.g., minimum node size) are met, producing a tree that maps predictors to outcomes through a series of interpretable decision rules. Although the CART algorithm evaluates many candidate partitions during tree construction, the final tree displays only the subset of splits that produce the largest reductions in within-node variance. Consequently, the resulting diagram summarises the most influential conditional relationships rather than enumerating all possible factor-level comparisons.

Balancing model flexibility with overfitting is a key aspect of tree modelling. The complexity parameter (CP) in *rpart* addresses this by penalising additional splits that do not sufficiently improve model fit [26,27]. Specifically, CP represents the minimum improvement in variance reduction required to justify a new split. Lower CP values permit deeper trees, while higher values prune the tree to a simpler structure. In EXCITE, the tree’s complexity parameter is directly linked to the statistical evidence from ANOVA. Specifically, the application automatically sets a CP threshold directly linked to the significance level of the ANOVA interaction terms. For example, when the ANOVA interaction is statistically significant at p < 0.01, EXCITE uses a lower CP threshold, permitting additional splits to reflect this interaction. In contrast, when interactions are nonsignificant, EXCITE applies a higher CP threshold to enforce stricter pruning. This approach ensures that the final tree prioritises splits aligned with statistically meaningful effects, reducing overfitting while preserving interpretability [13].

Additionally, EXCITE computes variable importance as the total variance reduction attributable to each predictor across all splits [27]. This measure highlights predictors that drive subgroup differentiation, even those not retained in the pruned tree, bridging machine-learning flexibility with traditional statistical rigour. For example, in a three-way ANOVA studying the effects of teaching method, school type, and gender on student performance, the variable ‘school type’ may be found to have high variable importance in the decision tree, even if it does not appear as a splitting variable in the final pruned tree. This could happen if ‘school type’ is strongly associated with ‘teaching method’, which is selected for splitting. As a result, ‘school type’ influences the structure of the interaction indirectly, highlighting its role despite its absence in the final displayed tree. By coupling this measure with interactive tree visualisations, EXCITE enables users to explore how key variables structure conditional subgroup patterns within the tree representation.

### 2.3. Mathematical specification of regression trees in EXCITE

In this section, we formalise the tree-building procedure implemented in the *rpart* package [28] as it is used in the EXCITE web application. Throughout, we follow the terminology and notation of Breiman et al [27].

Let


$$\begin{array}{c} D=\;{\left\{\left(y_{i},{\text{x}}_{i}\right)\right\}}_{i=\text{1}}^{n},\;{\text{x}}_{i}=\;\left(x_{i\text{1}},\cdots ,\;x_{ip}\right),\;{\;x}_{ij}\in \left\{\text{1},\;\cdots ,L_{j}\right\} \end{array}$$

denote *n* independent observations on a continuous response *y* and categorical predictors $x_{ij}$, each with $L_{j}$ unordered levels.

**Recursive binary partitioning.** A regression tree partitions the predictor space into *M* disjoint terminal regions $R_{\text{1}},\;\cdots ,\;R_{M}$. Beginning with the root node $R_{\text{1}}=\;X$, the algorithm considers all possible binary partitions of the levels for a given categorical predictor. For a predictor with $L_{j}$ levels, there are ${\text{2}}^{L_{j}-\text{1}}-\text{1}$ possible ways to form two groups. The algorithm applies binary splits of the form:


$$\begin{array}{c} R⟶\;\left\{ \begin{array}{c} R_{\text{L}}=\left\{\text{x }:\;x_{j}\in S\right\},\\ R_{\text{R}}=\left\{\text{x }:\;x_{j}\notin S\right\}, \end{array}\right. \end{array}$$

where $x_{j}$ is a categorical predictor and $S\subset \left\{\text{1},\;\cdots ,L_{j}\right\}$ is a non-empty proper subset of its levels.

For each candidate split *(j, S),* the *CART* cost function for continuous *y* is the *total within–node sum of squares* (i.e., the impurity of the region R)


$$\begin{array}{c} Q\left(R\right)=\sum_{i:{\text{x}}_{i}\in R}{\left(y_{i}-{\overset{―}{y}}_{R}\right)}^{\text{2}}\;,\;{\overset{―}{y}}_{R}={\left|R\right|}^{-\text{1}}\sum_{i:{\text{x}}_{i}\in R}y_{i} \end{array}$$

Choosing the split that maximises the reduction in impurity


$$\begin{array}{c} \Delta (j,\;S)=Q\left(R\right)-\;\left\{Q\left(R_{\text{L}}\right)+Q\left(R_{\text{R}}\right)\right\} \end{array}$$

is equivalent to minimising the residual sum of squares in the two child nodes.

This improvement can also be expressed as:


$$\begin{array}{c} \Delta (s,\;t)=\;\frac{n_{L}\times n_{R}}{n_{L}+n_{R}}\;\times {\left({\overset{―}{y}}_{L}-{\overset{―}{y}}_{R}\right)}^{\text{2}} \end{array}$$

where $n_{L}$ and $n_{R}\;$are the number of observations and ${\overset{―}{y}}_{L}$ and ${\overset{―}{y}}_{R}\;$are the means in the left and right child nodes, respectively.

Because all predictors are factors, Δ(j, S) is evaluated over every binary partition of the $L_{j}$ levels. For a predictor with $L_{j}\;$levels, an exhaustive enumeration requires ${\text{2}}^{L_{j}-\text{1}}-\text{1}$ subsets *S*.

*rpart* first orders the levels by their means and tests only the $L_{j}-\text{1}$ splits defined by that ordering, reducing the search to $O\left(L_{j}\text{log}L_{j}\right),\;$where O(·) is the big-O notation that gives an asymptotic upper bound on computational cost (up to constant factors).

**Cost–complexity pruning.** The fully grown tree $T_{\text{0}}$ over–fits, pruning produces a nested sequence $\left\{T_{\alpha }\right\}$ that balances bias and variance. For any subtree T, the cost–complexity functional [27] is:


$$\begin{array}{c} R_{\alpha }\left(T\right)=\;R\left(T\right)+\alpha \left|T\right|,\;R\left(T\right)=\sum_{m=\text{1}}^{\left|T\right|}n_{m}Q\left(R_{m}\right)\; \end{array}$$

where $n_{m}=\left|R_{m}\right|$ is the number of observations in leaf *m*, $Q\left(R_{m}\right)$ is the within-node mean-squared-error, so that $n_{m}Q\left(R_{m}\right)=SSE(R_{m})$, and |T| is the number of terminal nodes.

Increasing the complexity parameter α≥0 repeatedly removes the weakest link, the split with the smallest reduction in $\Delta /n_{m}$, and yields a nested sequence of sub-trees $T_{\alpha \text{1}}⊃T_{\alpha \text{2}}⊃\cdots .\;$The subtree selected for prediction balances goodness-of-fit (R(T)) against model parsimony (|T|). Therefore, cost-complexity pruning does not aim to discard scientifically meaningful effects but to avoid overfitting that may arise when recursive partitioning continues to split small subsets of data. The pruning step, therefore, prioritises stability and interpretability in the visualisation while retaining the most informative interaction structure.

**Tree selection in EXCITE.** The selection of the final regression tree is guided by both statistical evidence and interpretability. Let $p_{\text{int}}\;$denote the omnibus p-value of the highest-order interaction term from the preceding ANOVA model, representing the strength of evidence for the most complex interaction specified in the factorial ANOVA model. Rather than allowing users to adjust the tree depth arbitrarily, EXCITE links this statistical evidence directly to the tree’s complexity parameter (CP), the tuning constant that controls pruning in the CART framework.

Conceptually, EXCITE links the complexity of the decision tree directly to the statistical evidence provided by the factorial ANOVA. Stronger evidence for the highest-order interaction corresponds to a smaller complexity parameter (CP), allowing the tree to retain finer partitions of the data. Conversely, weaker evidence results in a larger CP value and consequently greater pruning, reducing the likelihood of displaying spurious interaction patterns. This adaptive mapping allows the visual complexity of the decision tree to reflect the strength of evidence for the highest-order interaction while promoting parsimonious and interpretable visualisations.

Practically, EXCITE provides two tree display modes that differ in the degree of pruning applied after tree construction: (i) *Maximum tree complexity*, which displays the fully grown tree (CP = 0), and (ii) *ANOVA-guided tree (recommended)*, which uses the omnibus p-value of the highest-order interaction ($p_{\text{int}}$) as the complexity parameter (CP).

A deep tree is first grown with CP = 0 using ten-fold cross-validation. Under the *Maximum tree complexity* option, this fully grown tree is displayed without additional pruning. Under the *ANOVA-guided tree* option, the tree is pruned using CP =$\;p_{\text{int}}\;$, thereby aligning the degree of pruning with the statistical evidence for the highest-order interaction. If the highest-order interaction is not statistically significant ($p_{\text{int}}\;$≥ 0.05), EXCITE suppresses the decision tree and informs the user that the ANOVA-guided tree is not displayed because there is insufficient evidence to support visualisation of the highest-order interaction.

These display modes do not alter the fitted ANOVA model or the interaction terms under consideration; rather, they control the visual complexity of the decision tree through the degree of pruning applied after tree construction. The relationship between the highest-order interaction p-value and the complexity parameter should therefore be interpreted as a heuristic mechanism for aligning visual model complexity with statistical evidence, rather than as a strict inferential rule.

This evidence-based approach formalises the principle that tree complexity should not exceed statistical support, translating inferential strength into visual model depth and reducing arbitrary parameter tuning. It bridges the inferential rigour of ANOVA with the exploratory flexibility of decision trees, enabling consistent and interpretable visualisations of interaction effects [26,27].

**Prediction.** Each terminal node carries the fitted value ${\hat{y}}_{m}={\overset{―}{y}}_{R_{m}}$, so that


$$\begin{array}{c} \hat{y}(\text{x})=\sum_{m=\text{1}}^{M}{\hat{y}}_{m}\text{1}\left\{\text{x}\in R_{m}\right\}, \end{array}$$

a piecewise-constant regression surface whose paths translate directly

into user-readable “if-then’‘ rules. That is, for a new observation **x**, we follow its decision path from the root to the appropriate terminal node and return the corresponding mean ${\hat{y}}_{m}$ as the prediction.

**Variable importance.** For a predictor $x_{j}$, its importance is the sum of the improvements over all nodes where $x_{j}\;$is the splitting variable, weighted by the proportion of observations reaching each node. The calculation is as follows:


$$\begin{array}{c} \text{VI}\left(x_{j}\right)=\;\sum_{t:x_{j}\text{ splits at }t}p(t)\times \Delta (s*,t) \end{array}$$

where the sum is over all internal nodes *t,* where $x_{j}\;$is the primary splitter, *p(t)* is the proportion of observations reaching node *t*, and Δ(s*,t) is the improvement from the spli*t*. This total contribution also includes the weighted improvement from surrogate splits that mimic the primary split. When predictors are strongly correlated, variable importance scores should be interpreted cautiously. Recursive partitioning algorithms may attribute variance to one predictor even when multiple correlated predictors contribute similarly to the observed pattern. Consequently, the variable importance measures in EXCITE should be interpreted as descriptive indicators of variance partitioning within the tree, rather than as providing a unique ranking of predictor importance.

### 2.4. Workflow and user interface overview

The EXCITE app visualises complex ANOVA interactions in a single plot, using insights from decision tree methodology. It is hosted freely on shinyapps.io at https://zq9mvv-vub0square.shinyapps.io/EXCITE-research-tool/. The reproducible source code containing a complete list of utilised R packages can be found on GitHub at https://github.com/vub-square/EXCITE-Shiny-app.

**Fig 2:**
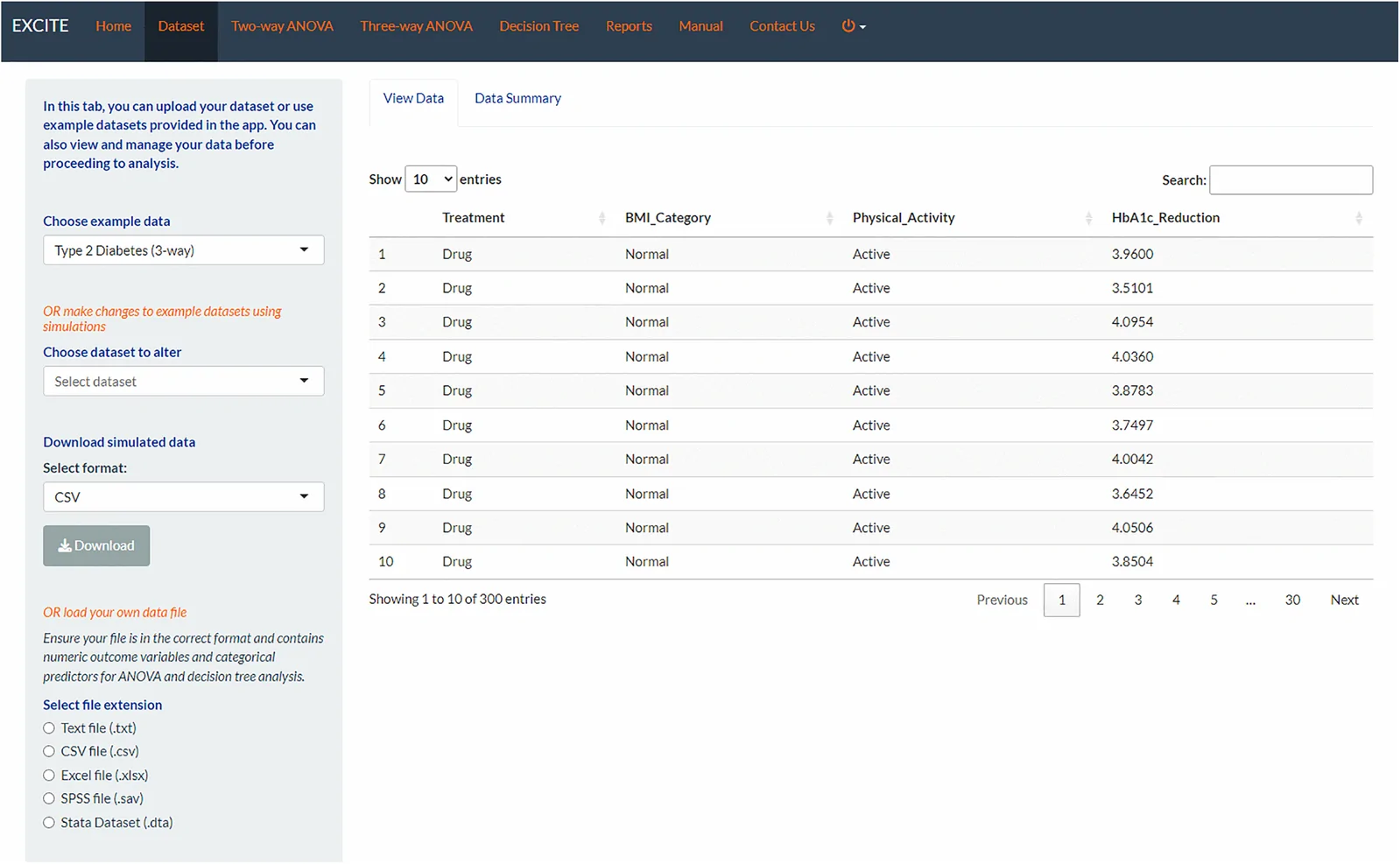
Dataset tab. The EXCITE app’s user interface displays a data preview.

The user interface comprises eight tabs: *Home, Dataset, Two-way ANOVA, Three-way ANOVA, Decision Tree, Reports, Manual,* and *Contact Us* (see Fig 2). The tabs consist of an input (sidebar) panel for the user inputs and an output (main) panel, where the outputs are displayed. The workflow of the app is structured into five interactive stages.

**Fig 3:**
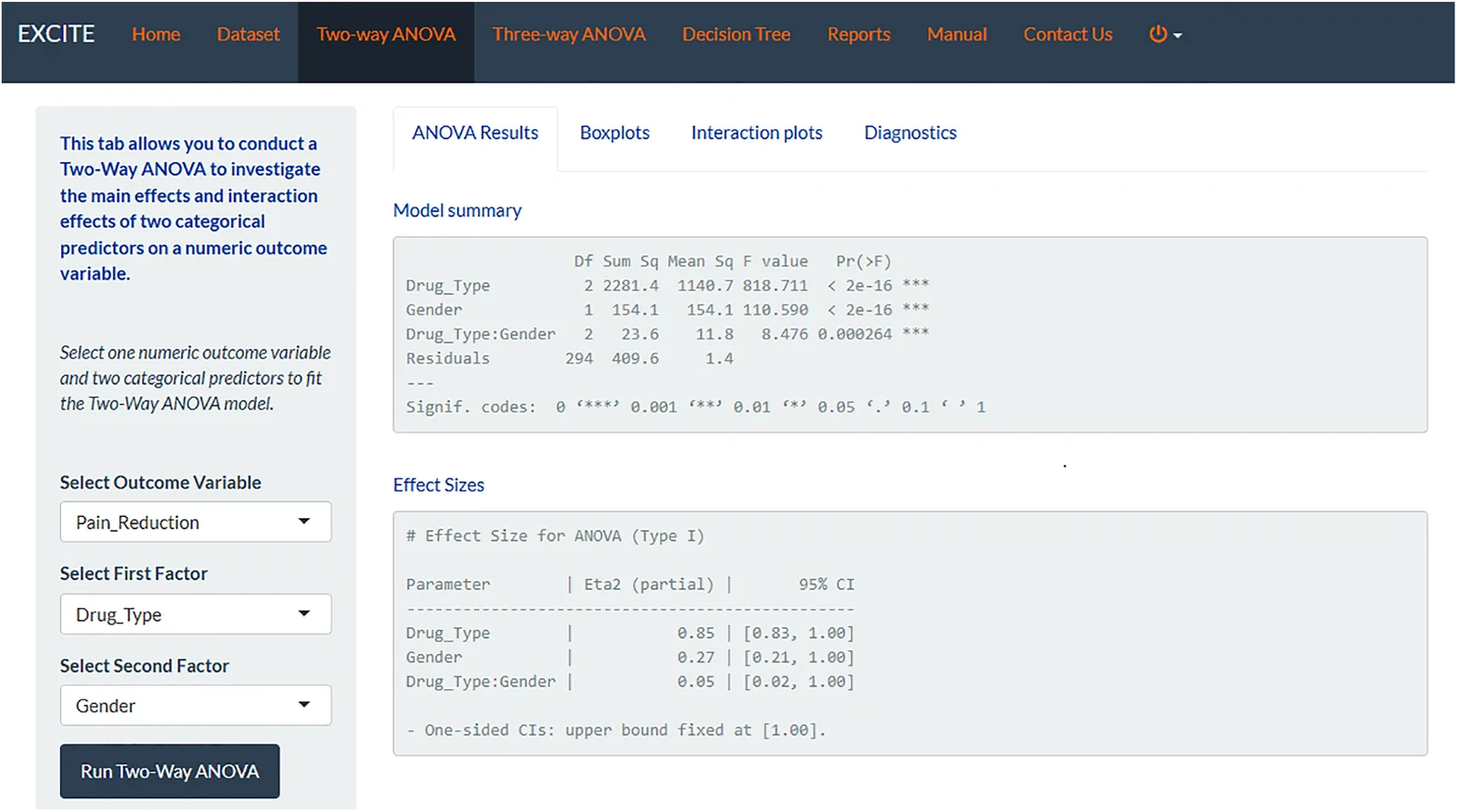
Two-way ANOVA tab. The main panel shows navigation to access ANOVA results, plots and diagnostics.

**Fig 4:**
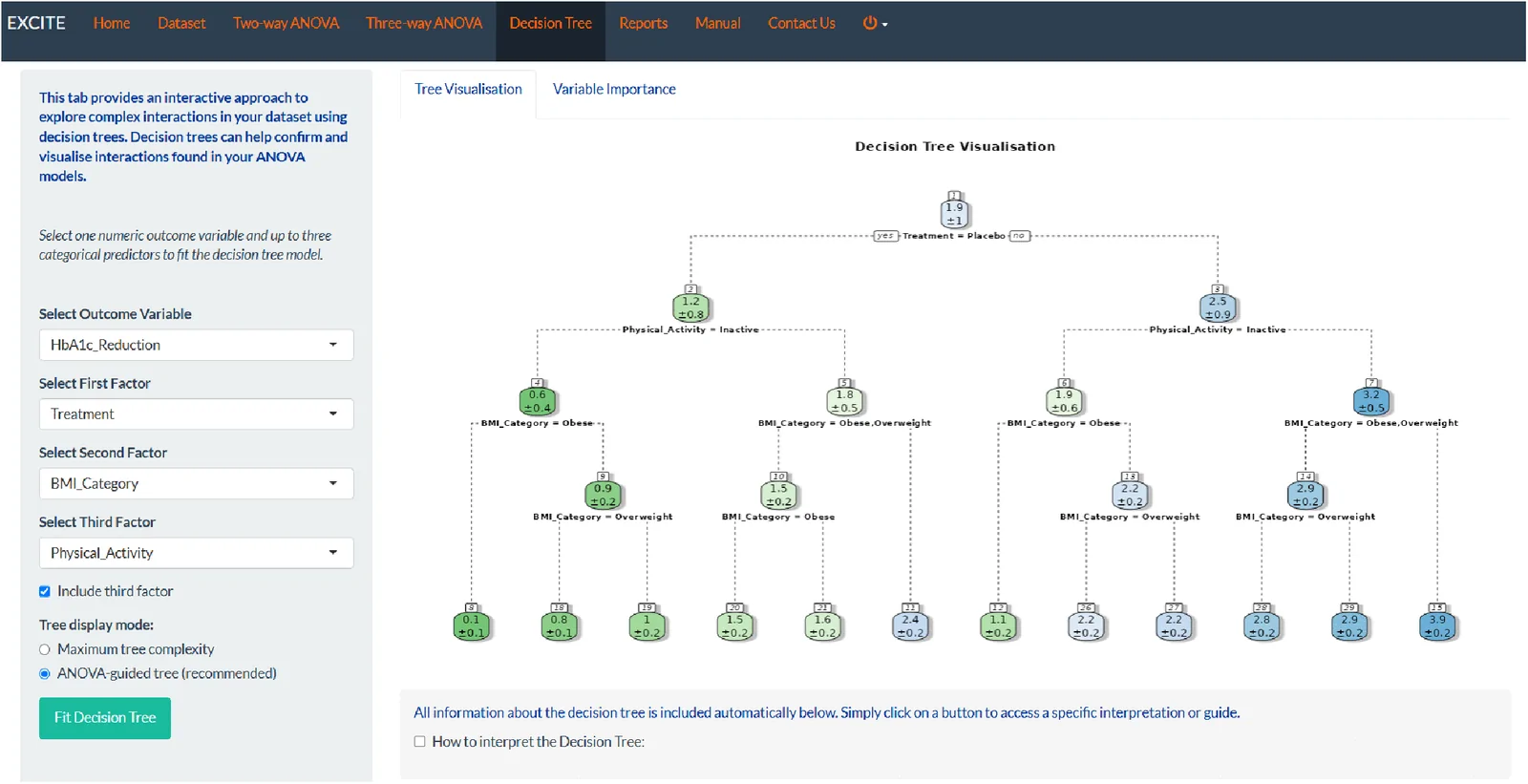
Decision Tree tab. The sidebar shows options to fit the tree model, and the main panel where the decision tree plot and summary will be displayed.

1. **Data Import**: On the *Dataset* tab (Fig 2), users begin by either uploading their datasets in various formats (CSV, Excel, SPSS, Stata, text) or selecting one of the example datasets. The application also allows users to simulate alternative versions of the built-in examples by manipulating two key design parameters: within-group variability and sample size, before any analysis is run. The latter can be done in the sidebar, under *“Choose dataset to alter”*, the user (i) chooses an example dataset (e.g., *“Pain reduction (2-way)”* or *“Type 2 Diabetes (3-way)”*), (ii) specifies a new standard deviation via a slider (thereby inflating or shrinking the residual variance), and (iii) sets the number of observations per factor combination with a numeric input (up to 200 per cell). Clicking “*Run Simulation”* generates a new dataset that honours the original factorial structure but reflects the requested variability and sample size. Adaptations in the dataset are immediately shown in the output of all downstream tabs, so users can explore how changes in confidence intervals’ width, sample size, or both influence ANOVA results and decision-tree visualisations. This feature turns the example datasets into interactive sandboxes for power and precision, rather than static demonstrations. A download button then allows the download of the simulated data in CSV, TSV, or Excel format for external use.
2. **Data Exploration and Management:** On the main panel of the *Dataset* tab, users can interactively preview the dataset in the *“View Data”* table, where they can sort, filter, and search rows using the table widgets as shown in Fig 2. The *“Data Summary”*, in the background generated with *summarytools* (v1.0.1), provides robust exploration tools that report summary statistics, detect variable types, and flag potential conversion issues. Under the *“Data manipulation and preprocessing”* heading on the sidebar panel, users can select variables to alter, for instance, change their data type (e.g., numeric to factor) or apply common transformations such as log, square root, standardisation, or centring. Clicking *“Apply Changes”* immediately writes the edited or newly transformed column back to the working dataset. This integrated workflow allows users to confirm that factor levels, numeric ranges, and missing-value patterns are sensible and, when needed, to fix them before proceeding to the ANOVA and Decision-Tree tabs.
3. **ANOVA Modelling**: Under the *Two-way ANOVA* and *Three-way ANOVA* tabs, users can perform an ANOVA analysis by selecting one numeric outcome variable and corresponding categorical predictor variables. The application then presents detailed model summaries (Fig 3), visualisations (including boxplots and interaction plots), and the ANOVA model diagnostics (e.g., Shapiro-Wilk normality test).
4. ***Decision Tree*** **Visualisation**: Building on the variables already specified for the ANOVA model, the main panel of the *Decision Tree* tab allows users to visualise the tree analysis. On the sidebar panel, users can select the tree display mode. The *Maximum tree complexity* option displays the fully grown tree, whereas the *ANOVA-guided tree (recommended)* prunes the tree using the omnibus p-value of the highest-order interaction as the complexity parameter (see Fig 4). The main panel displays two linked outputs: (i) an interactive “*Tree Visualisation”* and (ii) a bar plot under “*Variable Importance”*. The tree diagram shows, at each node, the mean outcome for that subgroup (top number) and its standard error (bottom number, preceded by “±”). To interpret a pathway in Fig 4, begin at the root node (top node), then move downward by selecting branches that match your factor levels. At each node, follow the left branch if the stated condition is met (“yes”) and the right branch if it is not (“no”). Continue this process until you reach a leaf node. The leaf node provides the mean outcome and its standard error (precision) for the specific combination of factor levels you’ve selected (e.g., “Placebo → Inactive → Obese BMI”). Smaller standard errors denote more precise estimates, while larger ones signal greater within-group variability. Every split visualises an ANOVA interaction, and the colour shading of the nodes reflects the predicted mean response for each terminal node. The green-blue gradient corresponds to the range of predicted values, with different colours indicating different levels of the outcome variable. This visual encoding allows for quick identification of subgroups with similar predicted outcomes, complementing the numerical predictions displayed at each node. The accompanying technical summary reports the CP table; EXCITE highlights the optimal CP that balances model fit and simplicity, so users can see why the displayed tree was pruned at a given depth. By embedding these explanations, accessible via checkbox “manual” buttons (e.g., ‘*How to interpret the Decision Tree’*), the interface gives step-by-step guidance on node values, standard errors, split logic, and pruning criteria, ensuring that researchers can interpret both the visual tree and the numerical summary with confidence.
5. **Reporting**: Under the *Reports* tab, EXCITE allows users to export detailed analysis reports in HTML, PDF, or Word formats, capturing the entire analytic workflow, including data summaries, ANOVA results, and decision tree visualisations.

## 3. Results

### 3.1. Example 1: Two-Way ANOVA

This example examines the effects of Drug Type (placebo, low dose, high dose) and Gender (male, female) on pain reduction in 300 simulated observations.

The two-way ANOVA results (Table 1 from the *Two-way ANOVA* tab in the app) show significant main effects of Drug Type (F(2, 294) = 818.71, p < .001) and Gender (F(1, 294) = 110.59, p < .001), and a significant Drug Type × Gender interaction (F(2, 294) = 8.48, p < .001). Effect sizes indicate a very large effect for Drug Type (η² = 0.85; 95% CI [0.83, 1.00]) and a moderate–large effect for Gender (η² = 0.27; [0.21, 1.00]); the interaction is small but significant (η² = 0.05; [0.02, 1.00]).

**Table 1:** Two-way ANOVA results for the effects of drug type and gender on pain reduction.

Source	*Df*	*SS*	*MS*	*F*	*p*	Partial η² (95% CI)
Drug Type	2	2281.4	1140.7	818.7	<.001	0.85 [0.83, 1.00]
Gender	1	154.1	154.1	110.6	<.001	0.27 [0.21, 1.00]
Drug Type × Gender	2	23.6	11.8	8.5	<.001	0.05 [0.02, 1.00]
Error (Residuals)	294	409.6	1.4			

Df = degrees of freedom; SS = sum of squares; MS = mean square; Partial η² = partial eta squared; CI = One-sided confidence intervals with upper bound fixed at 1.

**Fig 5:**
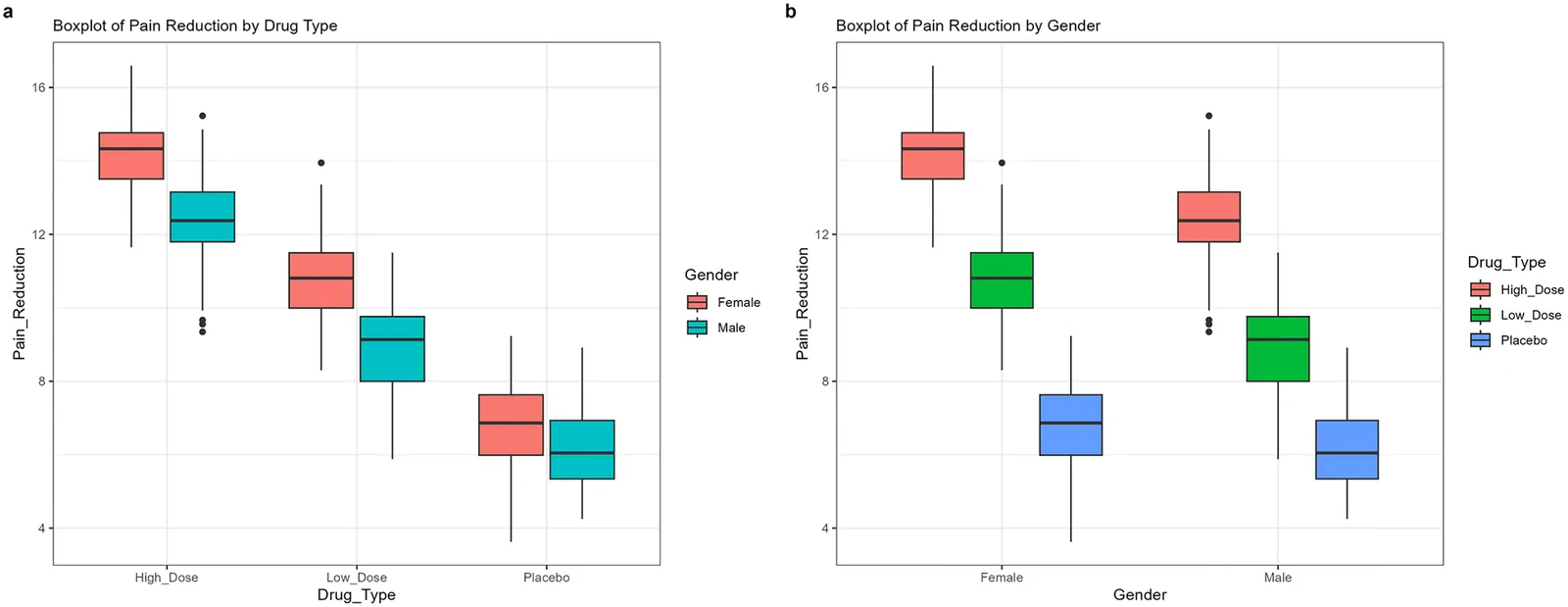
Boxplots from the Two-way ANOVA tab. (a) Pain reduction by drug type and (b) Pain reduction by gender.

**Fig 6:**
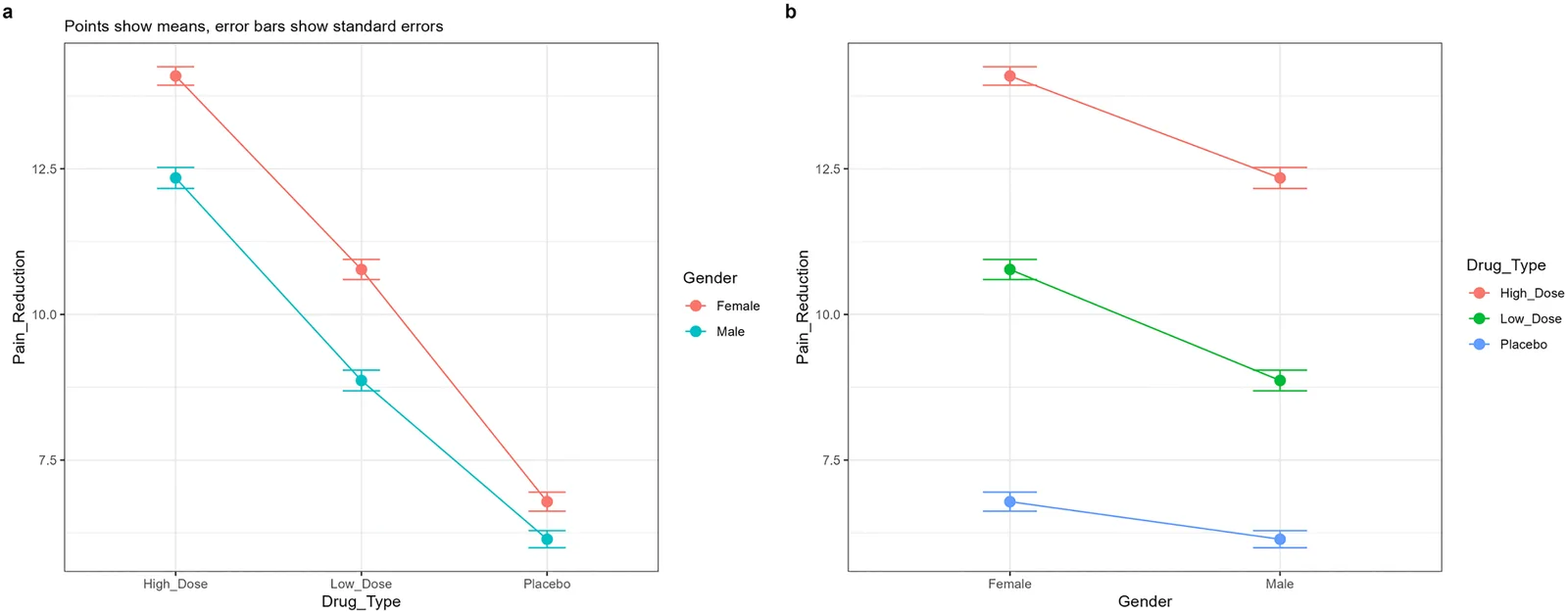
Interaction plots from the Two-way ANOVA tab. (a) Mean pain reduction by drug type and gender, and (b) Mean pain reduction by gender and drug type.

Fig 5 (boxplots) shows that pain reduction increases with higher dosage levels and is consistently higher for females. Fig 6 (interaction plots) illustrates the Drug Type × Gender interaction more clearly: slopes differ between genders, with females showing greater pain reduction across all dosages.

**Fig 7:**
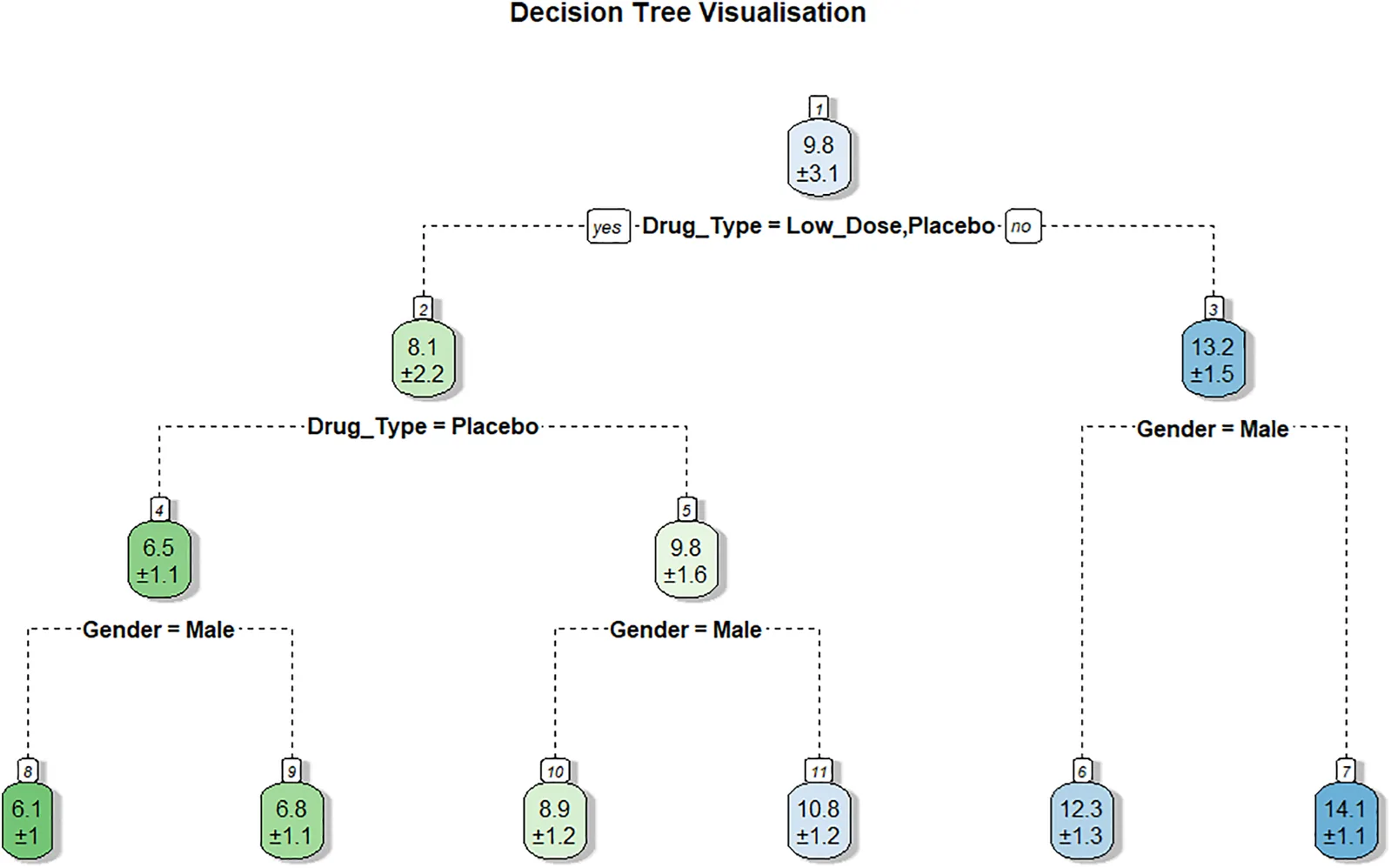
Two-way ANOVA decision tree visualisation of pain reduction. Illustrates recursive splitting based on drug type and gender.

The decision tree (Fig 7) highlights the conditional structure of this interaction. The root node sits at the top of the tree and contains every observation in the dataset; its mean of 9.8 is therefore the grand mean pain-reduction score across all treatment and gender combinations, and its accompanying standard error reflects the precision of this overall estimate. The first split is by Drug Type, separating high dose (node 3, mean = 13.2) from low/placebo (node 2, mean = 8.1). This is because a decision tree is restricted to binary splits; any factor with more than two levels is partitioned into two levels at a time; further downstream splits handle additional levels. Subsequent Gender splits show, for example, that within the high dose group, females (node 7, mean = 14.1) experience greater pain reduction than males (node 6, mean = 12.3). Similarly, in the placebo group, females (mean = 6.8) slightly outperform males (mean = 6.1), and in the low dose group, females (mean = 10.8) exceed males (mean = 8.9). Each node’s mean and standard error directly correspond to the subgroup averages shown earlier in boxplots and interaction plots, thus visually demonstrating how interactions influence subgroup differences within the tree representation.

Compared with Table 1 and Figs 5,6 (interaction plots and boxplots), the tree offers a single tree-based view of subgroup means, simplifying interpretation by showing how the main and interaction effects are characterised by successive tree splits.

### 3.2. Example 2: Three-way ANOVA (Social Feedback × Inclusion Condition × Fear of Negative Evaluation)

Whereas Example 1 illustrates how EXCITE visualises interactions between two categorical factors, this second example demonstrates the application of EXCITE to a three-way factorial design, where interpretation becomes substantially more challenging using conventional plots. Three-way ANOVA is commonly used when researchers seek to understand not only whether two factors interact, but whether that interaction itself depends on a third moderating variable. This setting provides a natural and practically relevant test case for evaluating whether a single, tree-based visualisation can clarify higher-order interaction structures.

To this end, we analyse real data from an online social interaction experiment reported by Thériault et al. [29], which examined how social feedback (ambiguous vs. positive) and inclusion condition (Cyberball vs. Uberball) jointly influence participants’ fundamental needs, and whether this relationship depends on individuals’ fear of negative evaluation (FNE). Fear of negative evaluation was dichotomised into Low FNE and High FNE, yielding a balanced 2x2x2 factorial design. This dataset is well-suited for illustrating three-way interactions, as prior analyses showed that the effect of social inclusion and feedback differs systematically across levels of social anxiety, producing interaction patterns that are difficult to synthesise using standard multi-panel visualisations.

Table 2 (from the Three-Way ANOVA tab) summarises the factorial structure underlying the patterns reported by Thériault et al. [29]. In the original study, the authors showed that individuals high in fear of negative evaluation are particularly sensitive to the combination of social feedback and inclusion context, with fundamental needs being lowest when positive feedback is followed by ordinary Cyberball inclusion. The present analysis reproduces this interaction structure in a standard three-way ANOVA framework to serve as input for visual exploration rather than as an independent test of new hypotheses.

**Table 2:** Three-way ANOVA results for the effects of feedback, inclusion condition, and fear of negative evaluation (FNE group) on fundamental needs.

Source	*Df*	*SS*	*MS*	*F*	*p*	Partial η² (95% CI)
Feedback	1	0.93	0.93	1.92	0.167	0.00 [0.00, 1.00]
Inclusion	1	10.21	10.2	21	<.001	0.05 [0.02, 1.00]
FNE	1	18.15	18.2	37.3	<.001	0.08 [0.04, 1.00]
Feedback × Inclusion	1	0.02	0.02	0.05	0.829	0.00 [0.00, 1.00]
Feedback × FNE	1	0.08	0.08	0.17	0.678	0.00 [0.00, 1.00]
Inclusion × FNE	1	0.7	0.7	1.43	0.233	0.00 [0.00, 1.00]
Feedback × Inclusion × FNE	1	4.48	4.48	9.2	0.003	0.02 [0.00, 1.00]
Error (Residuals)	430	209.4	0.49			

Df = degrees of freedom; SS = sum of squares; MS = mean square; Partial η² = partial eta squared; CI = One-sided confidence intervals with upper bound fixed at 1

**Fig 8:**
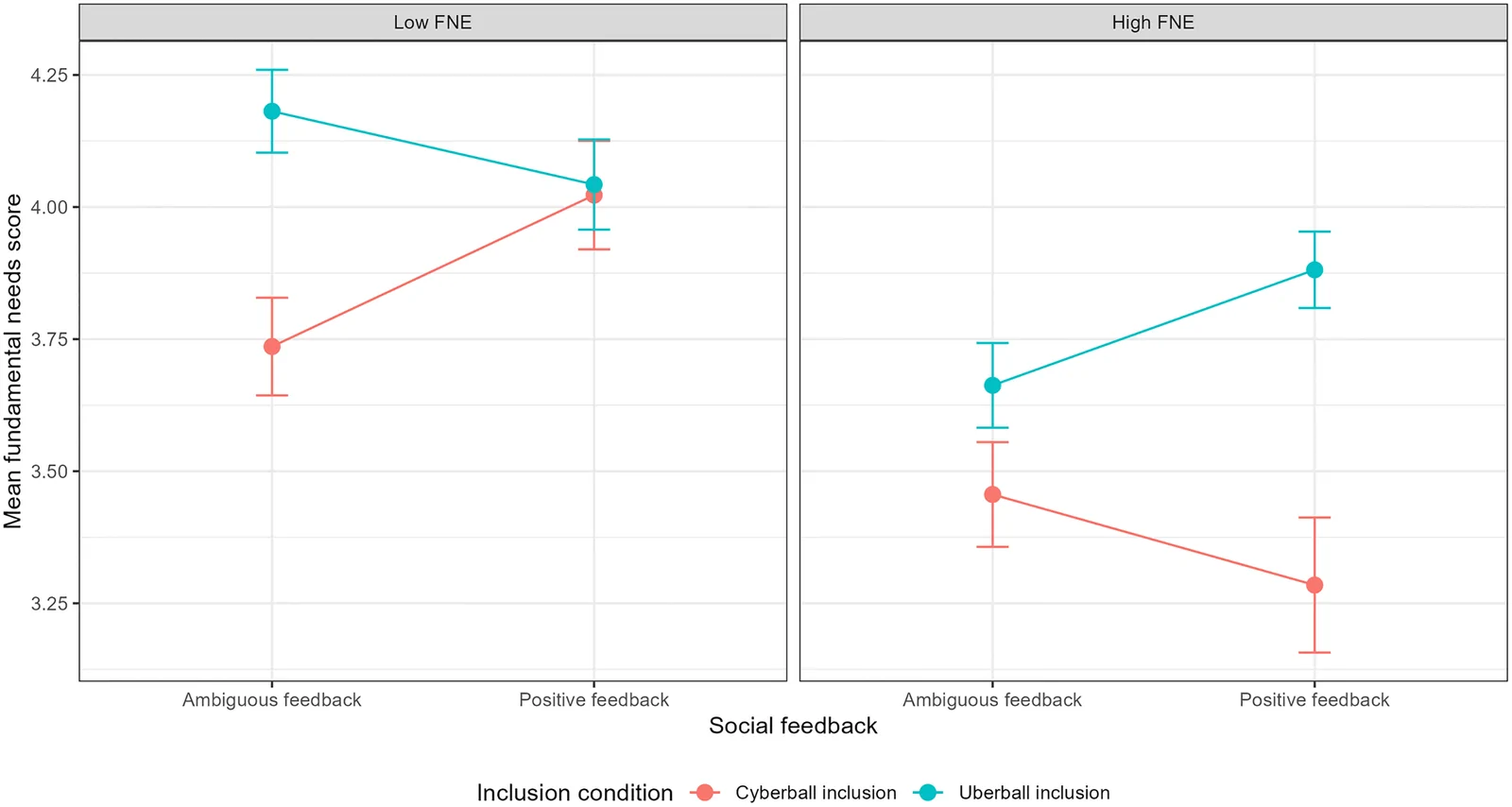
Interaction plots from the Three-way ANOVA tab. Plots showing feedback effects by inclusion condition and FNE group.

**Fig 9:**
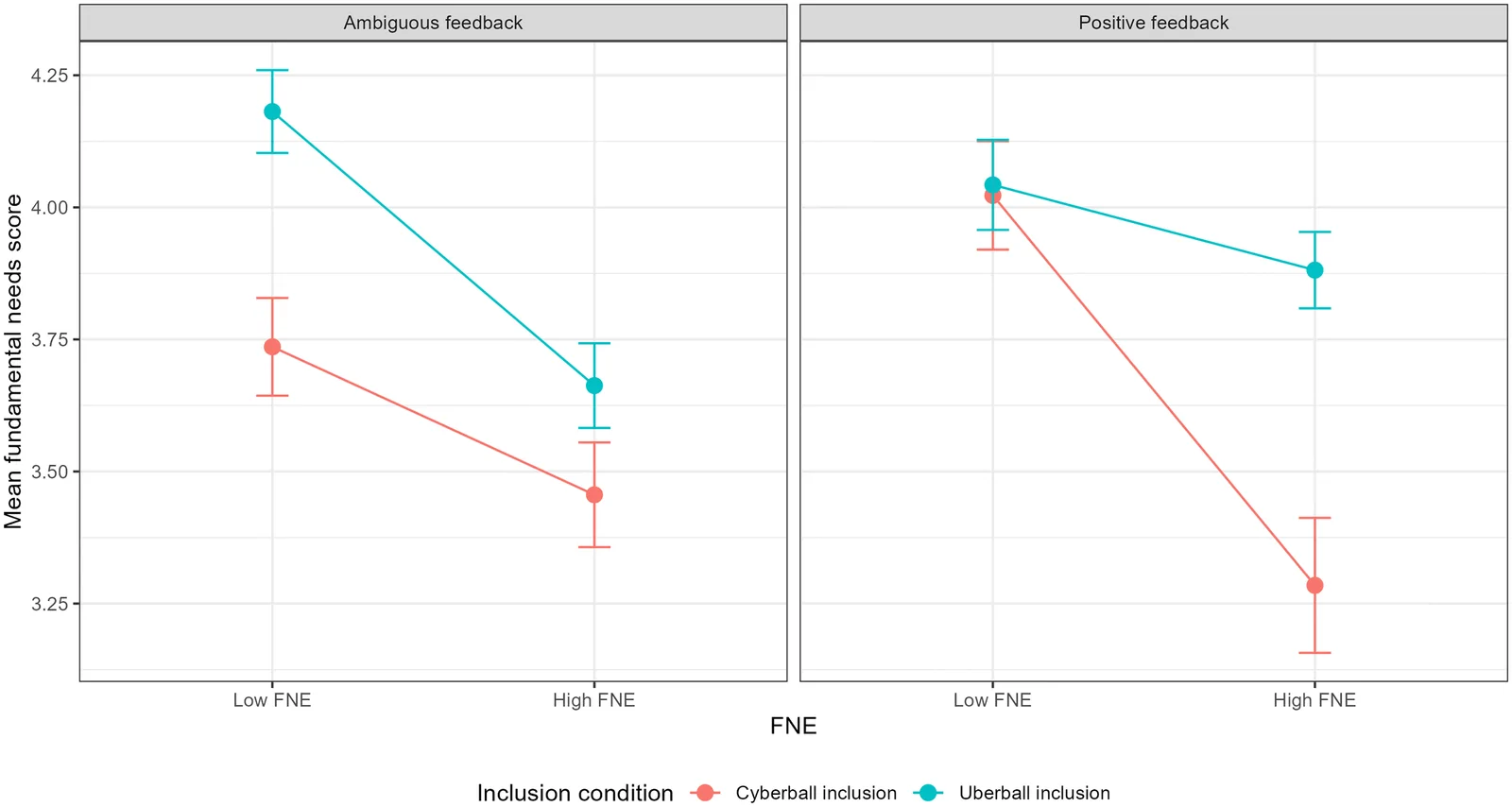
Interaction plots from the Three-way ANOVA tab. Plots showing inclusion effects by the FNE group across ambiguous and positive feedback conditions.

Consistent with the original findings, significant main effects were observed for Inclusion (F(1, 430) = 20.96, p < .001) and FNE group (F(1, 430) = 37.27, p < .001), alongside a statistically significant three-way interaction between Feedback, Inclusion, and FNE group (F(1, 430) = 9.20, p = .0026). The magnitude of this interaction was modest (partial η² ≈ 0.02), but its interpretive relevance lies in how conditional effects differ across levels of social anxiety, a pattern that becomes clearer when examined graphically. However, the confidence intervals for the effect-size estimates are wide, indicating considerable uncertainty in the magnitude of these effects, which is typical for higher-order interactions and should be taken into account when interpreting these results. Figs 8 and 9 closely mirror the interaction patterns described in the original study, but reorganise them to highlight conditional differences across FNE groups and inclusion contexts.

Fig 8 displays mean fundamental needs across feedback and inclusion conditions, separately for Low and High FNE. For Low FNE, needs scores were generally high across all conditions, with minor differences between feedback types. Uberball inclusion consistently produced higher means than Cyberball inclusion, but the magnitude of this difference was small. For High FNE, a clearer pattern emerged. Under Cyberball inclusion, participants who received positive feedback showed the lowest needs scores. In contrast, Uberball inclusion buffered this drop, yielding higher scores under both ambiguous and positive feedback. This demonstrates that individuals high in social anxiety are most sensitive to the combination of positive feedback followed by ordinary inclusion.

Fig 9 reorganises the results using feedback as the facetting variable, with the FNE group on the x-axis and lines representing the two inclusion conditions. Under Ambiguous feedback, both the Low and High FNE groups report higher fundamental needs following Uberball inclusion than Cyberball inclusion, with only a modest decline from Low to High FNE. Under Positive feedback, this pattern becomes more pronounced: High-FNE participants in the Cyberball condition show the lowest needs scores in the entire figure, whereas High-FNE participants in the Uberball condition maintain comparatively high needs. This layout illustrates that although Uberball inclusion benefits participants across FNE levels, it is especially protective for individuals with High FNE, who show substantial reductions in fundamental needs only when exposed to Positive feedback followed by Cyberball inclusion.

**Fig 10:**
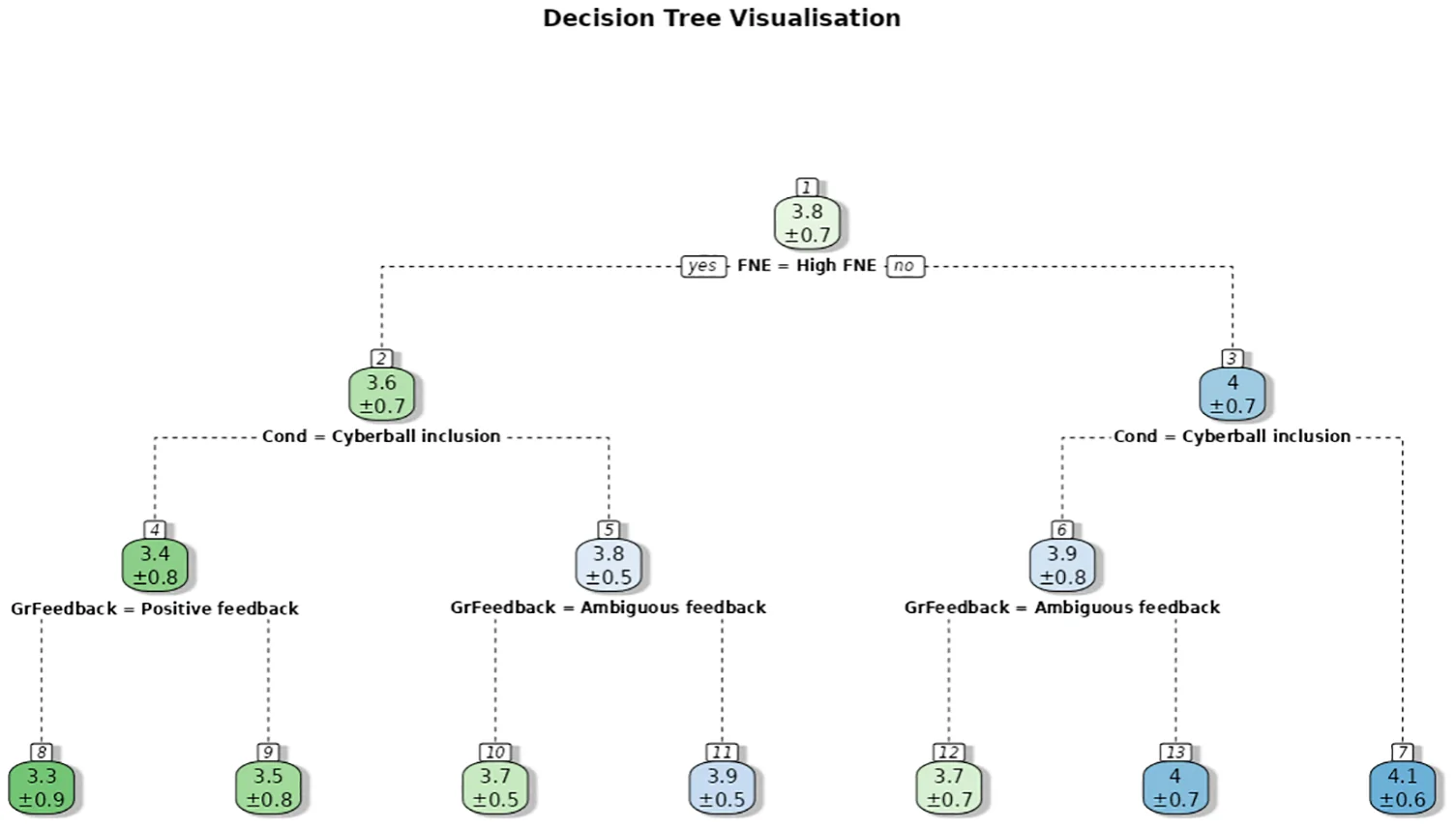
Three-way ANOVA decision tree visualisation of fundamental needs. Regression tree showing splits based on FNE group, inclusion condition, and feedback.

While Figs 8 and 9 illustrate the three-way structure through traditional interaction plots, each panel showing two factors at a time, this approach still requires readers to mentally integrate patterns across multiple subplots. The decision tree visualisation (Fig 10) condenses the same information into a single, unified tree structure. The root node splits first by FNE group, identifying social anxiety as the most influential predictor of fundamental needs. Participants with Low FNE form one branch with a comparatively high overall mean (node 3, M = 4.0 ± 0.7), whereas those with High FNE form a second branch with a lower mean (node 2, M = 3.6 ± 0.7).

Within the High FNE branch, the next split occurs on Inclusion condition, distinguishing the effects of Cyberball versus Uberball. High-FNE participants exposed to Cyberball (node 4) show reduced fundamental needs (M = 3.4 ± 0.8), particularly when the preceding feedback was positive (node 8, M = 3.3 ± 0.9), which represents the lowest outcome in the entire tree. In contrast, High-FNE individuals in the Uberball condition (node 5) report higher needs (M = 3.8 ± 0.5); among them, ambiguous feedback (node 10, M = 3.7 ± 0.5) maintains relatively stable scores, while positive feedback (node 11, M = 3.9 ± 0.5) yields the most favourable outcomes for this group.

In the Low FNE branch, the pattern is reversed in magnitude but consistent in structure. The next split again occurs on the Inclusion condition, with the Cyberball subgroup (node 6) showing somewhat lower needs (M = 3.9 ± 0.8), followed by a final split on feedback. Ambiguous feedback (node 12, M = 3.7 ± 0.7) produces slightly lower means than positive feedback (node 13, M = 4.0 ± 0.7). In contrast, Low-FNE participants receiving Uberball inclusion (node 7) show the highest needs overall (M = 4.1 ± 0.6), regardless of whether they received ambiguous or positive feedback beforehand.

Taken together, this tree-based representation (or single-plot strategy) mirrors and clarifies the patterns observed in Figs 8 and 9. By ordering predictors according to their relative contribution and displaying conditional effects along each decision path, the tree offers a single, integrated view of the three-way interaction, making complex subgroup differences more transparent and decision-oriented than a series of separate interaction plots.

**Fig 11:**
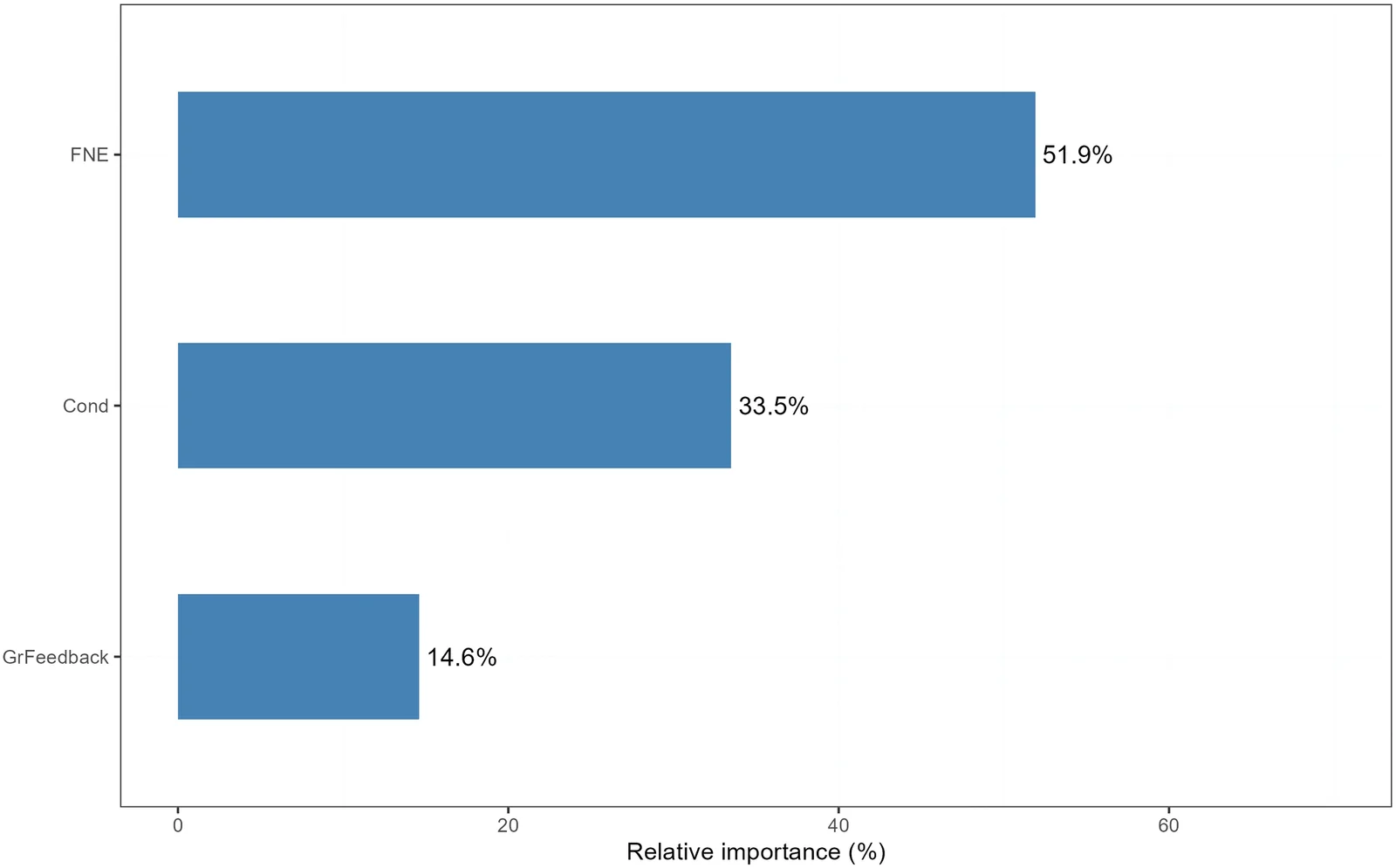
Variable importance in the Three-way ANOVA decision tree.

Fig 11 shows the variable importance fractions derived from the decision tree. FNE group contributed the largest proportion (51.9%), followed by Inclusion (33.5%), with Feedback contributing a smaller share (14.6%). This ranking matches the ANOVA: FNE and Inclusion exhibit the strongest main effects, while Feedback primarily plays a conditional role within the three-way interaction. The agreement between both approaches demonstrates the utility of EXCITE in revealing which variables matter most and how they interact.

## 4. Discussion

Decision trees are particularly well-suited for visualising ANOVA interactions because they provide an interpretable representation of conditional relationships between factors in a single diagram. Unlike traditional interaction plots that often require multiple separate visualisations, decision trees partition data recursively according to predictors that explain the largest reductions in within-node variance. The EXCITE Shiny application leverages this property by integrating decision-tree visualisations with factorial ANOVA analyses in an automated, user-guided workflow that takes researchers from data exploration to interpretation. This integration allows complex interactions, especially in three-way designs, to be displayed in one concise and interpretable graphic.

Importantly, the hierarchy shown in the decision tree should not be interpreted as implying that ANOVA interaction effects themselves are hierarchical. In factorial ANOVA, interactions represent simultaneous conditional relationships among factors. The hierarchy instead reflects the visual structure imposed by recursive partitioning, which orders conditional mean differences according to their contribution to variance reduction. In this sense, the decision tree provides an interpretable representation of conditional subgroup patterns rather than redefining the statistical structure of the interaction.

In this manuscript, we illustrate EXCITE using both simulated datasets and a real-world example drawn from a published social interaction study [29]. The empirical case demonstrates how the tool performs with naturally occurring variability and realistic interaction structures, while the simulated examples provide controlled settings in which the underlying interaction mechanisms are fully known. Together, these complementary data sources offer a balanced demonstration of EXCITE’s interpretive capabilities across both pedagogical and applied research contexts.

Other user-friendly statistical tools exist, and they either focus on automating assumption checks and appropriate test selection with customizable plots [14] or assist non-statisticians with introductory analyses such as one-way ANOVA [30]. In contrast, EXCITE is designed specifically to visualise complex factorial interactions within a single diagram, using the recursive partitioning nature of decision trees to reveal how factors jointly influence outcomes. This unified view mitigates the limitations of multiple conditional plots by presenting conditional subgroup differences in a single visual structure. However, EXCITE should be viewed as a complementary interpretive tool rather than a replacement for formal statistical inference. In particular, EXCITE does not replace post-hoc testing when specific contrasts must be evaluated. Instead, the tree representation helps researchers identify influential factor combinations that may warrant further statistical examination. When formal pairwise comparisons are conducted based on these patterns, appropriate multiplicity adjustments (e.g., Tukey, Bonferroni, or similar procedures) should be applied to control Type I error. By visually highlighting these conditional relationships, the tool can reduce the exploratory reliance on large numbers of pairwise comparisons while still allowing formal inferential procedures to be applied when needed. At the same time, researchers must recognise the trade-off between Type I and Type II errors when interpreting interaction effects. Post-hoc testing without adjustment inflates false positives, whereas corrections reduce power. By highlighting influential factor combinations directly, EXCITE helps researchers identify meaningful conditional patterns before conducting confirmatory statistical tests.

Regarding positioning relative to existing methods, EXCITE differs fundamentally from other visual machine-learning approaches like partial dependence plots and interaction decomposition models. Unlike these methods, which are designed for continuous predictors and predictive performance evaluation, EXCITE preserves the classical ANOVA context by mapping factor-level combinations through a tree-based representation. In this way, it serves as a complementary, ANOVA-coherent interpretive tool rather than a competing modelling framework.

Although decision trees complement traditional ANOVA analyses effectively, they also present statistical and software constraints. Statistically, trees can be unstable when datasets are small or unrepresentative of the underlying population, producing inconsistent splits across samples [26,27,31]. A further issue concerns surrogate splits, backup rules CART uses when predictors contain missing values [27]. Because all examples in this study used complete-case data, the app’s behaviour with missing values has not yet been validated. Additionally, when predictors are strongly correlated, recursive partitioning algorithms may attribute variance to one predictor even when multiple correlated predictors contribute to the observed pattern. Consequently, variable importance measures derived from decision trees should be interpreted as descriptive indicators of variance partitioning rather than definitive evidence of predictor importance. Standard ANOVA assumptions of normality, independence, and homoscedasticity also remain in effect; significant violations can compromise both ANOVA and tree accuracy without appropriate data transformation. Therefore, these assumptions should be verified before using the tool. In addition, when predictors are highly correlated, CART may preferentially attribute variable importance to one predictor over another without indicating that alternative correlated predictors provide comparable explanatory information. Accordingly, variable-importance measures should be interpreted with caution in the presence of multicollinearity, and users are encouraged to assess multicollinearity before interpreting tree-based results. EXCITE is intended as an interaction interpretation and visualisation tool for an already-specified ANOVA model rather than a comprehensive model-diagnostic platform.

From a software perspective, the current EXCITE version handles up to three-factor interactions and assumes basic ANOVA literacy from users. Moreover, the ANOVA-guided tree is conditioned on the statistical significance of the highest-order interaction and is therefore suppressed when this interaction is not significant, even if lower-order interactions remain significant. This design ensures consistency between the inferential evidence provided by the factorial ANOVA and the corresponding tree-based visualisation but limits the exploration of lower-order interactions in such situations. The current implementation also does not yet incorporate covariates as in ANCOVA.

The restriction to two- and three-factor ANOVA is intentional and reflects a principled first step in validating the integration of decision trees with classical factorial designs. Two- and three-way interactions represent the most commonly used experimental structures in applied research, and they provide a tractable setting in which to establish the correctness and interpretability of tree-based visualisation. While the decision-tree visualisation organises subgroup means in a hierarchical structure, the underlying statistical evidence remains grounded in the ANOVA framework presented alongside the tree output. Users should therefore interpret the tree as a visual summary of conditional relationships supported by the factorial model rather than as a substitute modelling framework. Extending EXCITE to four-way and higher-order interactions would introduce substantial visual complexity, making it difficult to rigorously test whether the approach enhances rather than hinders understanding. By first demonstrating that tree-based representations reliably capture and clarify lower-dimensional interactions, this work lays the methodological foundation for incorporating more complex designs in future versions.

A further limitation concerns the absence of direct empirical benchmarking against existing tools such as *moreThanANOVA*, partial dependence plots, or interaction decomposition models. Because EXCITE was developed as an interpretability companion to factorial ANOVA rather than a predictive or assumption-checking method, direct performance comparisons are not meaningful within the current framework. However, future evaluation will include a structured user study comparing EXCITE with multi-panel interaction plots and model-agnostic visualisations on interpretability outcomes, such as time-to-insight, accuracy in identifying the correct interaction pattern, and user confidence.

Future development should focus on improving model robustness and extending analytical scope. Because CART relies on surrogate splits for missing data, systematic benchmarking under realistic missing-data conditions, combined with user options to rank or disable surrogates, would increase reliability. Integrating ensemble algorithms such as random forests or gradient-boosted trees could stabilise predictions and variable-importance measures while preserving the existing interface. Another enhancement would be to allow the complexity parameter to adapt when higher-order interactions are non-significant, but lower-order ones remain important, thereby broadening pedagogical and applied value. Future developments could also explore alternative criteria for guiding tree complexity, such as effect-size, based or hybrid approaches, to complement the current p-value-guided heuristic. Expanding EXCITE to include covariates (ANCOVA) and higher-order or repeated-measures designs, alongside planned interpretability benchmarks with existing approaches, would further extend its usability. Such extensions would position EXCITE as a comprehensive, interaction-centred analytics platform rather than a single-tree extension of ANOVA.

## 5. Conclusion

EXCITE addresses a long-standing limitation in ANOVA-based research, the difficulty of interpreting complex interactions. By replacing multiple split-plot visualisations with a single, tree-based representation, the tool enables researchers to interpret multifactor interactions more intuitively and to recognise patterns that might otherwise remain obscured across separate plots. This integrated visualisation approach may help clarify the contribution of each factor combination and enhance understanding of higher-order interactions.

Beyond visualisation, EXCITE extends traditional ANOVA by pinpointing which factor-level combinations drive significant interactions. While omnibus F-tests merely signal the presence of an interaction, EXCITE’s decision-tree framework reveals where and how those effects occur, providing actionable insights rather than abstract significance. This targeted identification offers a principled alternative to conventional post-hoc pairwise comparisons, which often oversimplify complex effects and inflate Type I error through multiple testing.

By combining the inferential rigour of ANOVA with the interpretive precision of decision trees, EXCITE offers a practical and conceptually accessible framework for understanding multifactor relationships. The tool enhances statistical thinking by linking significance testing with visual exploration, empowering researchers across disciplines to interpret interactions more comprehensively and accurately. In doing so, EXCITE contributes to improving the quality and transparency of inference in experimental research and represents a step toward more intuitive, evidence-driven analytics in factorial designs. Taken together, these contributions position EXCITE as a complementary, ANOVA-coherent visualisation tool whose advantages will be further quantified through planned user-centred benchmarking focused on interpretability outcomes.
