# Supplementary material for: Integrating decision trees with ANOVA for enhanced interaction visualisations
Source: PLoS One. 2026 Sep 8;21(9):e0357663. doi: 10.1371/journal.pone.0357663 (PMC13552720; doi:10.1371/journal.pone.0357663)
Supplement: Supplementary file 1 [file pone.0357663.s001.pdf]

Review report of the manuscript "Integrating Decision Trees with ANOVA for Enhanced Interaction Visualisations"

The manuscript tackles an important and long-standing challenge in the statistical sciences: how to make complex ANOVA interactions, particularly three-way interactions, more interpretable. The proposed solution—EXCITE, an R Shiny application that combines traditional ANOVA models with decision tree visualisation—is conceptually appealing and aligns with ongoing efforts to create more accessible tools for applied researchers.

However, the paper falls short in several critical areas. First, the methodological innovation is not sufficiently substantiated. While decision trees are presented as a complement to ANOVA, the paper does not convincingly explain how this integration adds value beyond existing visualisation techniques, nor does it provide a strong theoretical justification for the chosen implementation (e.g., the mapping between p-values and tree complexity parameters). Second, the evaluation of EXCITE is inadequate. The examples provided are limited to simulated data, which reduces confidence in the tool's utility for real-world research contexts. Comparative analyses with established alternatives are missing, leaving the practical contribution of EXCITE unclear.

Moreover, the manuscript suffers from presentation issues that affect readability and impact. Figures are not always easy to interpret, the text includes frequent repetition, and references are inconsistently formatted. Collectively, these issues undermine the clarity of the work.

Strengths

1. The topic is timely and relevant, addressing recognised difficulties in interpreting higher-order ANOVA interactions.

2. The open-access R Shiny implementation has potential to be useful for researchers and educators.

Weaknesses

1. The integration of decision trees with ANOVA is only superficially explained. For example, the rationale for linking ANOVA p-values with the complexity parameter (CP) remains unclear and insufficiently justified.

2. The tool is not empirically compared with existing approaches (e.g., moreThanANOVA, partial dependence plots, interaction decomposition models). Without such benchmarks, it is difficult to assess the practical advantages of EXCITE.

3. The tool is restricted to two- and three-factor ANOVA designs. Although this limitation is acknowledged, the discussion of potential extensions is too superficial to compensate.

4. The examples rely solely on simulated datasets. No real-world case study is provided, which undermines the demonstration of practical applicability.

5. Figures (especially tree diagrams) are difficult to read and could be improved for clarity.

6. References contain inconsistencies and omissions.

7. The writing style is repetitive at times, which weakens the overall clarity of the manuscript.
