# Supplementary material for: Integrating decision trees with ANOVA for enhanced interaction visualisations
Source: PLoS One. 2026 Sep 8;21(9):e0357663. doi: 10.1371/journal.pone.0357663 (PMC13552720; doi:10.1371/journal.pone.0357663)
Supplement: Supplementary file 2 [file pone.0357663.s003.docx]

Iftikhar Ahmed Khan, Academic Editor

PLOS ONE

December 13, 2025

Dear Editor and Reviewer(s),

Thank you for taking the time to review our work (PONE-D-25-38509) and providing us with insightful feedback on ways to strengthen our paper. We have incorporated the suggested changes and addressed each point below.

We believe that the revised version better communicates the novelty and significance of our work. We are confident that our web application stands as a valuable addition to the existing body of knowledge in this field.

We hope that the revised version meets your expectations.

Thank you for your time and consideration.

On behalf of all the authors,

Yours sincerely,

Perseverence Savieri

Corresponding Author

Biostatistics and Medical Informatics Research Group (BISI)

Vrije Universiteit Brussel (VUB), Brussels 1090, Belgium

[Perseverence.Savieri@vub.ac.be](mailto:Perseverence.Savieri@vub.ac.be)

**Manuscript ID:** PONE-D-25-38509

**Manuscript title:** Integrating Decision Trees with ANOVA for Enhanced Interaction Visualisations

**Reviewer #1:**

*Weaknesses highlighted*

1. **The integration of decision trees with ANOVA is only superficially explained. For example, the rationale for linking ANOVA p-values with the complexity parameter (CP) remains unclear and insufficiently justified.**

We appreciate this insightful observation. We have expanded the explanation of how EXCITE links ANOVA p-values to the tree’s complexity parameter (CP) and clarified the conceptual rationale for this integration. Specifically, we now (i) provide a clearer justification that connects the strength of statistical evidence (p-value) to the permissible tree complexity, (ii) describe how this mapping maintains coherence between ANOVA inference and tree visualisation, and (iii) include supporting literature on cost-complexity pruning and model selection. These revisions appear in the Tree Selection in EXCITE subsection (page 12, lines 276-304).

2. **The tool is not empirically compared with existing approaches (e.g., moreThanANOVA, partial dependence plots, interaction decomposition models). Without such benchmarks, it is difficult to assess the practical advantages of EXCITE.**

We appreciate this important suggestion. Our primary objective in this manuscript is to introduce EXCITE as a single-view, interaction-centred visualisation companion to factorial ANOVA, rather than to propose a new estimator or a replacement for existing methods. Tools such as moreThanANOVA focus on assumption checks and post-hoc testing automation, while partial dependence plots (PDPs) and interaction decomposition models typically arise in machine-learning workflows and target different model classes (often continuous/coded predictors and flexible nonparametric fits). Because EXCITE is explicitly tied to factorial ANOVA with categorical factors and emphasises a one-diagram display of interaction structure, a direct “accuracy” or “predictive performance” benchmark is not the most informative comparison.

That said, we agree that positioning EXCITE against existing approaches will help readers understand its practical value. We have therefore (i) added a short positioning subsection that clarifies where EXCITE sits relative to moreThanANOVA, PDPs, and interaction decomposition, and (ii) expanded the limitations/future work to outline a planned user-study benchmarking interpretability outcomes (time-to-insight, accuracy of identifying the correct interaction pattern, and user confidence) across EXCITE vs. multi-panel interaction plots and PDP-style views. We believe these additions address the reviewer’s concern while keeping the paper focused on EXCITE’s intended role as an interpretability aid for factorial ANOVA.

3. **The tool is restricted to two- and three-factor ANOVA designs. Although this limitation is acknowledged, the discussion of potential extensions is too superficial to compensate.**

We thank the reviewer for raising this important point. Our intention in the present manuscript is to establish a clear and rigorous foundation for integrating decision-tree methodologies with classical ANOVA, with an emphasis on interpretability and methodological transparency. Because this work represents the first systematic step in exploring the use of decision trees to visualise ANOVA interactions, we deliberately restricted the implementation to two- and three-factor designs. This decision was made for two main reasons:

*Conceptual clarity and methodological validation.*

Two- and three-way interactions represent the most commonly used factorial designs in applied research, and they provide a tractable setting in which to test, validate, and illustrate how hierarchical tree structures can meaningfully and accurately represent ANOVA interactions. Introducing higher-order interactions (four-way and above) without first establishing interpretability and correctness in lower-dimensional settings would risk confounding the methodological contribution.

*Interpretability considerations.*

One of EXCITE’s core aims is to reduce the burden associated with interpreting multi-factor interactions. While decision trees can technically accommodate higher-order splits, the resulting trees quickly become unwieldy and visually dense. Beginning with two- and three-factor designs allows us to demonstrate the interpretive advantages of the approach before extending it to settings where visual complexity naturally increases.

We agree that outlining future extensions is valuable, and we have therefore expanded the Discussion to articulate a clearer development roadmap. This includes extending EXCITE to support (i) four-way and higher-order interactions, (ii) ANCOVA models with covariates, and (iii) the use of ensemble tree methods to improve robustness at higher dimensionality. These additions help to position EXCITE as an initial, principled foundation for a broader class of tree-based visualisation tools for factorial designs.

4. **The examples rely solely on simulated datasets. No real-world case study is provided, which undermines the demonstration of practical applicability.**

We thank the reviewer for this helpful observation. In the revised manuscript, we have now added a real-world case study based on data from a study on an online social interaction experiment (Thériault et al., 2023). This newly added analysis, presented in Section 3.2, demonstrates how EXCITE performs on empirical data containing naturally occurring variability, non-ideal balance, and realistic interaction patterns. Including this example directly addresses the reviewer’s concern by showing EXCITE’s applicability to actual research scenarios.

At the same time, we maintain that the simulation examples remain an important and deliberate component of the manuscript. Simulated datasets allow us to (i) clearly illustrate the mechanics of EXCITE in controlled settings where the underlying interaction structure is known, (ii) demonstrate how the built-in simulation module can alter noise levels, sample sizes, and effect patterns to mirror real-world conditions, and (iii) ensure reproducibility when explaining methodological features of the tool. This dual-example strategy, using both controlled simulations and a real dataset, enables readers to see EXCITE applied in transparent, pedagogical settings as well as in an authentic applied context. The combination of real and simulated examples, therefore, provides a balanced and comprehensive demonstration of the tool’s practical usability. We have also updated the Discussion to reflect this enhancement.

5. **Figures (especially tree diagrams) are difficult to read and could be improved for clarity.**

We thank the reviewer for this valuable observation. In the revised manuscript, all figures, including the decision tree diagrams, have been updated to improve clarity, consistency, and visual accessibility. We corrected and reformatted the plots using the journal’s PACE-compliant figure guidelines, ensuring improved readability, higher resolution, and clearer labelling across the tree diagrams, interaction plots, and ANOVA visualisations. We believe these revisions substantially enhance the interpretability and overall presentation quality of the figures.

6. **References contain inconsistencies and omissions.**

We thank the reviewer for noting the inconsistencies in the reference list. Although the manuscript was originally prepared using Mendeley, we have now conducted a full, manual audit of all references. We verified each entry against the source, corrected incomplete information (e.g., missing page ranges, inconsistent publisher details, and truncated DOIs), resolved duplicates, and ensured consistent formatting of book titles and journal names in accordance with the journal’s reference style. We are confident that the revised reference list is now complete and fully consistent.

7. **The writing style is repetitive at times, which weakens the overall clarity of the manuscript.**

We thank the reviewer for this valuable feedback. We have carefully reviewed the entire manuscript to eliminate redundancy and improve clarity. Specifically, we revised sentences that repeated definitions or concepts across sections (e.g., reiterations of ANOVA fundamentals, decision tree principles, and descriptions of the app workflow) and consolidated overlapping explanations. We also streamlined transitional phrases and restructured several paragraphs for smoother logical flow between sections. These revisions have shortened the text, reduced duplication, and improved readability while preserving all essential technical details. Changes were implemented throughout the Methods (Sections 2.1-2.4), Results (Examples 1 and 2), and Discussion sections.
