# Supplementary material for: Integrating decision trees with ANOVA for enhanced interaction visualisations
Source: PLoS One. 2026 Sep 8;21(9):e0357663. doi: 10.1371/journal.pone.0357663 (PMC13552720; doi:10.1371/journal.pone.0357663)
Supplement: Supplementary file 3 [file pone.0357663.s004.docx]

Iftikhar Ahmed Khan, Academic Editor

PLOS ONE

March 25, 2026

Dear Editor and Reviewer(s),

Thank you for taking the time to review our work (PONE-D-25-38509R1) and providing us with insightful feedback on ways to strengthen our paper. We have incorporated the suggested changes and addressed each point below.

We believe that the revised version better communicates the novelty and significance of our work. We are confident that our web application stands as a valuable addition to the existing body of knowledge in this field.

We hope that the revised version meets your expectations.

Thank you for your time and consideration.

On behalf of all the authors,

Yours sincerely,

Perseverence Savieri

Corresponding Author

Biostatistics and Medical Informatics Research Group (BISI)

Vrije Universiteit Brussel (VUB), Brussels 1090, Belgium

[Perseverence.Savieri@vub.ac.be](mailto:Perseverence.Savieri@vub.ac.be)

[perseverencesavieri@gmail.com](mailto:perseverencesavieri@gmail.com)

**Manuscript ID:** PONE-D-25-38509R1

**Manuscript title:** Integrating Decision Trees with ANOVA for Enhanced Interaction Visualisations

**Response to Reviewer #4:**

We thank the reviewer for the thoughtful and methodologically engaged comments. You raise several conceptual points regarding the interpretation of interactions and the positioning of the EXCITE framework. We have revised the manuscript to clarify these aspects. Importantly, EXCITE is designed as a visualisation and interpretation tool that complements classical ANOVA, rather than replacing its inferential framework. Below, we address each comment in detail.

***Major comments***

**Comment 1: Interactions do not have a hierarchical structure**

We agree with the reviewer that ANOVA interaction terms are not hierarchical. Interactions represent simultaneous conditional relationships among factors, and our intention was not to imply otherwise. Instead, the hierarchy arises from the visual representation imposed by recursive partitioning, which orders conditional mean differences according to their contribution to variance reduction. In other words, the hierarchy in the trees reflects the structure of the tree visualisation, not the statistical structure of the interaction itself. You are right that we should make this very clear.
Nonetheless, tree-based representations of interactions are widely used in statistical learning because they provide an interpretable representation of conditional relationships (Breiman et al., 1984; Hastie et al., 2009). In this context, our trees should be interpreted as a visual ordering of conditional effects, analogous to the way interaction plots organise effects across panels. To avoid possible misunderstanding, we clarified this explicitly in the manuscript (lines 174-176) and revised several occurrences of the term “hierarchical” so that it refers only to the structure of the tree representation rather than to the interaction itself.

**Comment 2: The method appears to remove the need for post-hoc testing.**

We appreciate the opportunity to clarify this point. EXCITE does not replace formal post-hoc inference, and this was not our intention. Rather, EXCITE addresses a common practical issue: researchers often conduct many exploratory pairwise comparisons to identify interaction patterns, and these comparisons can proliferate rapidly as the number of factor levels increases. The decision tree provides a data-driven visual decomposition of conditional means, allowing researchers to identify influential factor combinations before conducting confirmatory contrasts. However, this exploratory step does not eliminate the need for formal statistical inference. When specific contrasts are tested, appropriate adjustments for multiple comparisons (e.g., Bonferroni, Tukey, or similar procedures) remain necessary to control Type I error. To avoid ambiguity, we have revised the manuscript to state that EXCITE complements explicitly, but does not replace, post-hoc inference, and that multiplicity correction remains essential when evaluating statistical significance (lines 609–612).

**Comment 3: You write that EXCITE highlights the influential combinations directly, but the algorithm is checking all the combinations too, isn't it?**

We appreciate this clarification request. The reviewer is correct that the CART algorithm evaluates many possible candidate splits internally when constructing the regression tree. In that sense, the algorithm implicitly considers multiple possible partitions of the factor levels. However, the purpose of the decision-tree representation in EXCITE is not to enumerate all possible comparisons but to identify and display the partitions that produce the largest reductions in within-node variance, as defined by the CART splitting criterion (Breiman et al., 1984). In other words, although many candidate combinations are evaluated during tree construction, the final visualisation presents only the subset of conditional splits that most strongly structure the response variable. This behaviour differs from manually examining all cell means or conducting numerous pairwise comparisons, where every contrast must be inspected separately and, importantly, requires appropriate correction for multiple testing. The decision tree, therefore, provides a structured summary of the most influential conditional relationships, which can guide subsequent confirmatory analyses. When formal comparisons are conducted based on these patterns, standard multiplicity adjustments remain necessary to ensure valid statistical inference. To clarify this point, we added a short explanation in the manuscript (lines 183-187 and lines 618-631).

**Comment 4: The method relies heavily on p-values when tuning the complexity parameter.**

The reviewer raises an important point regarding the interpretation of p-values. In EXCITE, the p-value is used only as a heuristic to guide tree complexity, not as a strict inferential criterion. Specifically, the mapping between interaction significance and the complexity parameter aligns the depth of the tree visualisation with the strength of the statistical evidence. This approach prevents overly complex trees when interaction evidence is weak, while still allowing deeper partitions when interactions are strongly supported. Importantly, the ANOVA output displayed in the application includes effect sizes (partial η²) alongside p-values, and the interpretation of results should always consider both. We have clarified this point in the manuscript (lines 286-289). With respect to the reviewer’s concern about the typically low statistical power of interaction effects, it is important to note that the pruning rule affects only the visual tree representation and does not alter the underlying factorial ANOVA results. All estimated interaction terms remain available in the statistical output, allowing researchers to investigate higher-order effects directly, even when the tree representation is simplified for interpretability. The pruning rule therefore serves to stabilise the visualisation and reduce over-interpretation of small or unstable subgroup splits, rather than discouraging careful examination of interaction effects. We agree that alternative criteria, such as effect-size–based thresholds, could provide a valuable complement to p-value–guided pruning. The current implementation represents a first, pragmatic approach aligned with conventional ANOVA outputs, but future versions of EXCITE could incorporate effect-size–driven or hybrid criteria to guide tree complexity. We have added this as a limitation and direction for future development in the manuscript (lines 695-697).

**Comment 5: The multicollinearity example may imply that the algorithm identifies the most influential variable.**

We agree with the reviewer that when predictors are strongly correlated, recursive partitioning methods, like regression models, cannot reliably distinguish which variable is most influential. In such cases, the allocation of variance to a particular predictor depends on the structure of the data and the splitting criterion, and different but highly correlated predictors may lead to similar partitions. The purpose of the example was to illustrate that variable importance measures can reflect how predictors contribute to variance partitioning within the tree, even if they are not selected as splitting variables in the final pruned tree. However, we acknowledge that in the presence of multicollinearity, these measures should not be interpreted as uniquely identifying the most important predictor. We have revised the manuscript to clarify that variable importance reflects the role of predictors within the tree representation and should be interpreted with caution when predictors are correlated (lines 309–315).

**Comment 6: Why use cost-complexity pruning if higher-order interactions are scientifically meaningful?**

Cost-complexity pruning is a standard component of CART methodology (Breiman et al., 1984) and is used to prevent overfitting caused by recursive splitting of small subsets. The goal in EXCITE is not to suppress meaningful interactions but to ensure that the tree visualisation remains stable and interpretable. Without pruning, regression trees often grow deep structures that reflect sampling noise rather than meaningful patterns. Importantly, the ANOVA results remain fully available in the interface, meaning that all estimated interaction terms remain accessible even when the visual tree is pruned (lines 261-265).

**Comment 7: Is it problematic that interactions require time to interpret?**

We agree that complex interactions often require careful interpretation. The aim of EXCITE is not to oversimplify statistical reasoning but to provide a visual framework that helps researchers organise conditional relationships more efficiently. In many applied settings, interaction plots across multiple panels can be difficult to synthesise visually. The tree representation provides a complementary view that consolidates these conditional relationships in a single diagram.

**Comment 8: Concern about the three-way ANOVA example and wide effect-size intervals.**

We thank the reviewer for this important observation. We agree that the wide confidence intervals for the partial η² estimates indicate that the effect sizes are estimated with considerable uncertainty, making it difficult to distinguish precisely between small and larger effects based on these estimates alone. This imprecision likely reflects the limited information available for estimating higher-order interaction effects in the dataset, a common issue in factorial designs where interaction effects tend to have lower statistical power and greater variability than main effects.

The purpose of this example is not to emphasise precise effect-size estimation, but to illustrate how EXCITE visualises conditional interaction structures in a realistic applied setting. Importantly, the interaction pattern itself is consistent with the findings reported in the original study (Thériault et al., 2023), and the decision tree representation focuses on the relative structure of subgroup differences rather than the magnitude of effect sizes. To address the reviewer’s concern, we have revised the manuscript to explicitly acknowledge the uncertainty in the effect-size estimates and clarify that the example is intended for visualisation purposes rather than precise effect-size interpretation (lines 507-510).

**Comment 9: n-way ANOVA notation is unnecessary and adds rather little to the content.**

We agree and have removed the general n-way notation to improve clarity.

**Comment 10: Suggestion regarding wording on higher-order interactions**

While we appreciate the reviewer’s humour, we felt that adopting this wording might introduce an unintended tone. However, we agree with the underlying point that higher-order interactions should not be omitted purely for convenience. To reflect this, we have revised the manuscript to emphasise that while higher-order interactions are often excluded to simplify interpretation or preserve statistical power, model specification should be guided by theoretical considerations and study design rather than convenience alone. This clarification has been added in the Methods section (lines 148–150).

***Minor corrections***

- All typographical issues identified by the reviewer were corrected.
- *Figure 10 clarification*: The reviewer suggested a possible labelling error in Figure 10. We carefully checked the figure and confirmed that the labels are correct. No changes were required.

We thank the reviewer again for the constructive comments, which helped improve the clarity and conceptual positioning of the manuscript.
