# Supplementary material for: Integrating decision trees with ANOVA for enhanced interaction visualisations
Source: PLoS One. 2026 Sep 8;21(9):e0357663. doi: 10.1371/journal.pone.0357663 (PMC13552720; doi:10.1371/journal.pone.0357663)
Supplement: Supplementary file 4 [file pone.0357663.s005.docx]

Iftikhar Ahmed Khan, Academic Editor

PLOS ONE

August 12, 2026

Dear Editor and Reviewer(s),

Thank you for taking the time to review our work (PONE-D-25-38509R2) and providing us with insightful feedback on ways to strengthen our paper. We have incorporated the suggested changes and addressed each point below.

We believe that the revised version better communicates the novelty and significance of our work. We are confident that our web application stands as a valuable addition to the existing body of knowledge in this field.

We hope that the revised version meets your expectations.

Thank you for your time and consideration.

On behalf of all the authors,

Yours sincerely,

Perseverence Savieri

Corresponding Author

Biostatistics and Medical Informatics Research Group (BISI)

Vrije Universiteit Brussel (VUB), Brussels 1090, Belgium

[Perseverence.Savieri@vub.ac.be](mailto:Perseverence.Savieri@vub.ac.be)

[perseverencesavieri@gmail.com](mailto:perseverencesavieri@gmail.com)

**Manuscript ID:** PONE-D-25-38509R2

**Manuscript title:** Integrating Decision Trees with ANOVA for Enhanced Interaction Visualisations

**Reviewer #4:**

*Weaknesses highlighted*

**Comment 1: Clarification of the definition of** $\boldsymbol{p}_{\mathbf{int}}$

We thank the reviewer for identifying this ambiguity. The implementation of EXCITE does not use the smallest interaction p-value across all interaction terms. Rather, it uses the p-value corresponding to the highest-order interaction term (i.e., the two-way interaction in a two-factor ANOVA or the three-way interaction in a three-factor ANOVA) to determine the complexity parameter. We have therefore revised the manuscript (lines 269-272) to remove the phrase "smallest omnibus interaction p-value" and instead explicitly state that $p_{\mathrm{int}}$ denotes the omnibus p-value for the highest-order interaction. This wording more accurately reflects both the methodology and software implementation.

**Comment 2: Clarification of tree complexity options**

Thank you for this helpful observation. We agree that the previous label "Model interactions" was ambiguous and did not accurately reflect the functionality of EXCITE. The application always fits the complete factorial ANOVA model; the available options control only how the resulting decision tree is displayed and pruned, rather than which interaction terms are included in the ANOVA model. To address this, we simplified the user interface by replacing the previous interaction labels with two tree display modes: *Maximum tree complexity* and *ANOVA-guided tree (recommended)*. We also revised the corresponding manuscript text (lines 284-304 and 390-394) to clarify that these options control the degree of pruning applied to the decision tree while leaving the underlying ANOVA model unchanged.

**Comment 3: Wording describing interaction effects**

We thank the reviewer for this helpful suggestion. We agree that the term "unfold" could inadvertently imply a sequential process rather than the simultaneous conditional relationships represented by ANOVA interactions. We have revised the wording accordingly (lines 476-477) to clarify that the successive tree splits provide a hierarchical representation of the conditional structure of the fitted ANOVA model rather than suggesting that interaction effects develop sequentially.

**Comment 4: Handling multicollinearity in EXCITE**

We appreciate the reviewer's suggestion. We agree that multicollinearity can influence CART variable-importance measures and may not be readily recognised by inexperienced users. However, EXCITE was intentionally designed as an interaction interpretation and visualisation tool that operates after a factorial ANOVA model has been specified, rather than as a comprehensive model-building or diagnostic platform. Incorporating multicollinearity diagnostics would substantially broaden the scope of the application beyond its intended purpose and would logically require the inclusion of other model diagnostics, such as checks for normality, homoscedasticity, independence, and influential observations. Instead, we have strengthened the discussion of this limitation in the manuscript (lines 673-680) by explicitly advising users to assess multicollinearity and other model assumptions prior to interpreting the decision tree and its variable-importance measures.
