# Supplementary material for: Integrating decision trees with ANOVA for enhanced interaction visualisations
Source: PLoS One. 2026 Sep 8;21(9):e0357663. doi: 10.1371/journal.pone.0357663 (PMC13552720; doi:10.1371/journal.pone.0357663)
Supplement: Peer review history

# Peer review: Decision Letter 0

**DOI:** 10.1371/journal.pone.0357663.r001

**Authors:** Iftikhar Khan

11 Sep 2025

Dear Dr. Savieri,

Thank you for submitting your manuscript to PLOS ONE. After careful consideration, we feel that it has merit but does not fully meet PLOS ONE’s publication criteria as it currently stands. Therefore, we invite you to submit a revised version of the manuscript that addresses the points raised during the review process.

Please submit your revised manuscript by Oct 26 2025 11:59PM. If you will need more time than this to complete your revisions, please reply to this message or contact the journal office at plosone@plos.org. When you're ready to submit your revision, log on to https://www.editorialmanager.com/pone/ and select the 'Submissions Needing Revision' folder to locate your manuscript file.

- A rebuttal letter that responds to each point raised by the academic editor and reviewer(s). You should upload this letter as a separate file labeled 'Response to Reviewers'.
- A marked-up copy of your manuscript that highlights changes made to the original version. You should upload this as a separate file labeled 'Revised Manuscript with Track Changes'.
- An unmarked version of your revised paper without tracked changes. You should upload this as a separate file labeled 'Manuscript'.

If you would like to make changes to your financial disclosure, please include your updated statement in your cover letter. Guidelines for resubmitting your figure files are available below the reviewer comments at the end of this letter.

If applicable, we recommend that you deposit your laboratory protocols in protocols.io to enhance the reproducibility of your results. Protocols.io assigns your protocol its own identifier (DOI) so that it can be cited independently in the future. For instructions see: https://journals.plos.org/plosone/s/submission-guidelines#loc-laboratory-protocols. Additionally, PLOS ONE offers an option for publishing peer-reviewed Lab Protocol articles, which describe protocols hosted on protocols.io. Read more information on sharing protocols at https://plos.org/protocols?utm_medium=editorial-email&utm_source=authorletters&utm_campaign=protocols.

We look forward to receiving your revised manuscript.

Kind regards,

Iftikhar Ahmed Khan

Academic Editor

PLOS ONE

**Journal Requirements:**

1. When submitting your revision, we need you to address these additional requirements.

Please ensure that your manuscript meets PLOS ONE's style requirements, including those for file naming. The PLOS ONE style templates can be found at

https://journals.plos.org/plosone/s/file?id=wjVg/PLOSOne_formatting_sample_main_body.pdf and

https://journals.plos.org/plosone/s/file?id=ba62/PLOSOne_formatting_sample_title_authors_affiliations.pdf

2. We note that your Data Availability Statement is currently as follows: All relevant data are within the manuscript and its supporting information files.

Please confirm at this time whether or not your submission contains all raw data required to replicate the results of your study. Authors must share the “minimal data set” for their submission. PLOS defines the minimal data set to consist of the data required to replicate all study findings reported in the article, as well as related metadata and methods (https://journals.plos.org/plosone/s/data-availability#loc-minimal-data-set-definition).

For example, authors should submit the following data:

- The values behind the means, standard deviations and other measures reported;

- The values used to build graphs;

- The points extracted from images for analysis.

Authors do not need to submit their entire data set if only a portion of the data was used in the reported study.

If your submission does not contain these data, please either upload them as Supporting Information files or deposit them to a stable, public repository and provide us with the relevant URLs, DOIs, or accession numbers. For a list of recommended repositories, please see https://journals.plos.org/plosone/s/recommended-repositories.

If there are ethical or legal restrictions on sharing a de-identified data set, please explain them in detail (e.g., data contain potentially sensitive information, data are owned by a third-party organization, etc.) and who has imposed them (e.g., an ethics committee). Please also provide contact information for a data access committee, ethics committee, or other institutional body to which data requests may be sent. If data are owned by a third party, please indicate how others may request data access.

3. If the reviewer comments include a recommendation to cite specific previously published works, please review and evaluate these publications to determine whether they are relevant and should be cited. There is no requirement to cite these works unless the editor has indicated otherwise. 

**Additional Editor Comments:**

Please see the attached comment file.

[Note: HTML markup is below. Please do not edit.]

Reviewers' comments:

Reviewer's Responses to Questions

**Comments to the Author**

1. Is the manuscript technically sound, and do the data support the conclusions?

Reviewer #1: Partly

Reviewer #2: Yes

**********

2. Has the statistical analysis been performed appropriately and rigorously?

Reviewer #1: No

Reviewer #2: Yes

**********

3. Have the authors made all data underlying the findings in their manuscript fully available?

The PLOS Data policy

Reviewer #1: Yes

Reviewer #2: Yes

**********

4. Is the manuscript presented in an intelligible fashion and written in standard English?

Reviewer #1: Yes

Reviewer #2: Yes

**********

**Reviewer #1:**  Please see my comments attached.

Please see my comments attached.

(Sorry for this)

**Reviewer #2:**  The manuscript is technically sound and the analysis is well designed. Creating visualization techniques for interpretation effects in ANOVA is highly challenging. Looking at the analysis and methodology presented in the article, it seems an excellent way to present visualization of interpretation effects in. a multi variable ANOVA. The authors have explained the methodology using examples very well. They also explained the mathematical reasoning quite well so that anyone with a little knowledge can understand it clearly. The examples help understanding the complex analysis presented in a simple manner making it easy for non mathematicians to understand as well.

**********

what does this mean?). If published, this will include your full peer review and any attached files.

If you choose “no”, your identity will remain anonymous but your review may still be made public.

**Do you want your identity to be public for this peer review?**  For information about this choice, including consent withdrawal, please see our Privacy Policy

Reviewer #1: No

Reviewer #2: **Yes:** Priyadarsini Dasari

**********

[NOTE: If reviewer comments were submitted as an attachment file, they will be attached to this email and accessible via the submission site. Please log into your account, locate the manuscript record, and check for the action link "View Attachments". If this link does not appear, there are no attachment files.]

While revising your submission, please upload your figure files to the Preflight Analysis and Conversion Engine (PACE) digital diagnostic tool, https://pacev2.apexcovantage.com/. PACE helps ensure that figures meet PLOS requirements. To use PACE, you must first register as a user. Registration is free. Then, login and navigate to the UPLOAD tab, where you will find detailed instructions on how to use the tool. If you encounter any issues or have any questions when using PACE, please email PLOS at figures@plos.org. Please note that Supporting Information files do not need this step.

Attachment Submitted filename: Review report of the manuscript.pdf

# Author comment: Author response to Decision Letter 1

**DOI:** 10.1371/journal.pone.0357663.r002

16 Dec 2025

Manuscript ID: PONE-D-25-38509

Manuscript title: Integrating Decision Trees with ANOVA for Enhanced Interaction Visualisations

Reviewer #1:

Weaknesses highlighted

1. The integration of decision trees with ANOVA is only superficially explained. For example, the rationale for linking ANOVA p-values with the complexity parameter (CP) remains unclear and insufficiently justified.

We appreciate this insightful observation. We have expanded the explanation of how EXCITE links ANOVA p-values to the tree’s complexity parameter (CP) and clarified the conceptual rationale for this integration. Specifically, we now (i) provide a clearer justification that connects the strength of statistical evidence (p-value) to the permissible tree complexity, (ii) describe how this mapping maintains coherence between ANOVA inference and tree visualisation, and (iii) include supporting literature on cost-complexity pruning and model selection. These revisions appear in the Tree Selection in EXCITE subsection (page 12, lines 276-304).

2. The tool is not empirically compared with existing approaches (e.g., moreThanANOVA, partial dependence plots, interaction decomposition models). Without such benchmarks, it is difficult to assess the practical advantages of EXCITE.

We appreciate this important suggestion. Our primary objective in this manuscript is to introduce EXCITE as a single-view, interaction-centred visualisation companion to factorial ANOVA, rather than to propose a new estimator or a replacement for existing methods. Tools such as moreThanANOVA focus on assumption checks and post-hoc testing automation, while partial dependence plots (PDPs) and interaction decomposition models typically arise in machine-learning workflows and target different model classes (often continuous/coded predictors and flexible nonparametric fits). Because EXCITE is explicitly tied to factorial ANOVA with categorical factors and emphasises a one-diagram display of interaction structure, a direct “accuracy” or “predictive performance” benchmark is not the most informative comparison.

That said, we agree that positioning EXCITE against existing approaches will help readers understand its practical value. We have therefore (i) added a short positioning subsection that clarifies where EXCITE sits relative to moreThanANOVA, PDPs, and interaction decomposition, and (ii) expanded the limitations/future work to outline a planned user-study benchmarking interpretability outcomes (time-to-insight, accuracy of identifying the correct interaction pattern, and user confidence) across EXCITE vs. multi-panel interaction plots and PDP-style views. We believe these additions address the reviewer’s concern while keeping the paper focused on EXCITE’s intended role as an interpretability aid for factorial ANOVA.

3. The tool is restricted to two- and three-factor ANOVA designs. Although this limitation is acknowledged, the discussion of potential extensions is too superficial to compensate.

We thank the reviewer for raising this important point. Our intention in the present manuscript is to establish a clear and rigorous foundation for integrating decision-tree methodologies with classical ANOVA, with an emphasis on interpretability and methodological transparency. Because this work represents the first systematic step in exploring the use of decision trees to visualise ANOVA interactions, we deliberately restricted the implementation to two- and three-factor designs. This decision was made for two main reasons:

Conceptual clarity and methodological validation.

Two- and three-way interactions represent the most commonly used factorial designs in applied research, and they provide a tractable setting in which to test, validate, and illustrate how hierarchical tree structures can meaningfully and accurately represent ANOVA interactions. Introducing higher-order interactions (four-way and above) without first establishing interpretability and correctness in lower-dimensional settings would risk confounding the methodological contribution.

Interpretability considerations.

One of EXCITE’s core aims is to reduce the burden associated with interpreting multi-factor interactions. While decision trees can technically accommodate higher-order splits, the resulting trees quickly become unwieldy and visually dense. Beginning with two- and three-factor designs allows us to demonstrate the interpretive advantages of the approach before extending it to settings where visual complexity naturally increases.

We agree that outlining future extensions is valuable, and we have therefore expanded the Discussion to articulate a clearer development roadmap. This includes extending EXCITE to support (i) four-way and higher-order interactions, (ii) ANCOVA models with covariates, and (iii) the use of ensemble tree methods to improve robustness at higher dimensionality. These additions help to position EXCITE as an initial, principled foundation for a broader class of tree-based visualisation tools for factorial designs.

4. The examples rely solely on simulated datasets. No real-world case study is provided, which undermines the demonstration of practical applicability.

We thank the reviewer for this helpful observation. In the revised manuscript, we have now added a real-world case study based on data from a study on an online social interaction experiment (Thériault et al., 2023). This newly added analysis, presented in Section 3.2, demonstrates how EXCITE performs on empirical data containing naturally occurring variability, non-ideal balance, and realistic interaction patterns. Including this example directly addresses the reviewer’s concern by showing EXCITE’s applicability to actual research scenarios.

At the same time, we maintain that the simulation examples remain an important and deliberate component of the manuscript. Simulated datasets allow us to (i) clearly illustrate the mechanics of EXCITE in controlled settings where the underlying interaction structure is known, (ii) demonstrate how the built-in simulation module can alter noise levels, sample sizes, and effect patterns to mirror real-world conditions, and (iii) ensure reproducibility when explaining methodological features of the tool. This dual-example strategy, using both controlled simulations and a real dataset, enables readers to see EXCITE applied in transparent, pedagogical settings as well as in an authentic applied context. The combination of real and simulated examples, therefore, provides a balanced and comprehensive demonstration of the tool’s practical usability. We have also updated the Discussion to reflect this enhancement.

5. Figures (especially tree diagrams) are difficult to read and could be improved for clarity.

We thank the reviewer for this valuable observation. In the revised manuscript, all figures, including the decision tree diagrams, have been updated to improve clarity, consistency, and visual accessibility. We corrected and reformatted the plots using the journal’s PACE-compliant figure guidelines, ensuring improved readability, higher resolution, and clearer labelling across the tree diagrams, interaction plots, and ANOVA visualisations. We believe these revisions substantially enhance the interpretability and overall presentation quality of the figures.

6. References contain inconsistencies and omissions.

We thank the reviewer for noting the inconsistencies in the reference list. Although the manuscript was originally prepared using Mendeley, we have now conducted a full, manual audit of all references. We verified each entry against the source, corrected incomplete information (e.g., missing page ranges, inconsistent publisher details, and truncated DOIs), resolved duplicates, and ensured consistent formatting of book titles and journal names in accordance with the journal’s reference style. We are confident that the revised reference list is now complete and fully consistent.

7. The writing style is repetitive at times, which weakens the overall clarity of the manuscript.

We thank the reviewer for this valuable feedback. We have carefully reviewed the entire manuscript to eliminate redundancy and improve clarity. Specifically, we revised sentences that repeated definitions or concepts across sections (e.g., reiterations of ANOVA fundamentals, decision tree principles, and descriptions of the app workflow) and consolidated overlapping explanations. We also streamlined transitional phrases and restructured several paragraphs for smoother logical flow between sections. These revisions have shortened the text, reduced duplication, and improved readability while preserving all essential technical details. Changes were implemented throughout the Methods (Sections 2.1-2.4), Results (Examples 1 and 2), and Discussion sections.

Attachment Submitted filename: Response to Reviewers.docx

# Peer review: Decision Letter 1

**DOI:** 10.1371/journal.pone.0357663.r003

**Authors:** Iftikhar Khan

15 Feb 2026

Dear Dr. Savieri,

Thank you for submitting your manuscript to PLOS ONE. After careful consideration, we feel that it has merit but does not fully meet PLOS ONE’s publication criteria as it currently stands. Therefore, we invite you to submit a revised version of the manuscript that addresses the points raised during the review process.

Please submit your revised manuscript by Apr 01 2026 11:59PM. If you will need more time than this to complete your revisions, please reply to this message or contact the journal office at plosone@plos.org. When you're ready to submit your revision, log on to https://www.editorialmanager.com/pone/ and select the 'Submissions Needing Revision' folder to locate your manuscript file.

- A letter that responds to each point raised by the academic editor and reviewer(s). You should upload this letter as a separate file labeled 'Response to Reviewers'.
- A marked-up copy of your manuscript that highlights changes made to the original version. You should upload this as a separate file labeled 'Revised Manuscript with Track Changes'.
- An unmarked version of your revised paper without tracked changes. You should upload this as a separate file labeled 'Manuscript'.

If you would like to make changes to your financial disclosure, please include your updated statement in your cover letter. Guidelines for resubmitting your figure files are available below the reviewer comments at the end of this letter.

If applicable, we recommend that you deposit your laboratory protocols in protocols.io to enhance the reproducibility of your results. Protocols.io assigns your protocol its own identifier (DOI) so that it can be cited independently in the future. For instructions see: https://journals.plos.org/plosone/s/submission-guidelines#loc-laboratory-protocols. Additionally, PLOS ONE offers an option for publishing peer-reviewed Lab Protocol articles, which describe protocols hosted on protocols.io. Read more information on sharing protocols at https://plos.org/protocols?utm_medium=editorial-email&utm_source=authorletters&utm_campaign=protocols.

We look forward to receiving your revised manuscript.

Kind regards,

Iftikhar Ahmed Khan

Academic Editor

PLOS One

Journal Requirements:

If the reviewer comments include a recommendation to cite specific previously published works, please review and evaluate these publications to determine whether they are relevant and should be cited. There is no requirement to cite these works unless the editor has indicated otherwise.

[Note: HTML markup is below. Please do not edit.]

Reviewers' comments:

Reviewer's Responses to Questions

**Comments to the Author**

Reviewer #3: All comments have been addressed

Reviewer #4: (No Response)

**********

2. Is the manuscript technically sound, and do the data support the conclusions?

Reviewer #3: Yes

Reviewer #4: Partly

**********

3. Has the statistical analysis been performed appropriately and rigorously?

Reviewer #3: Yes

Reviewer #4: No

**********

4. Have the authors made all data underlying the findings in their manuscript fully available?

The PLOS Data policy

Reviewer #3: Yes

Reviewer #4: Yes

**********

5. Is the manuscript presented in an intelligible fashion and written in standard English?

Reviewer #3: Yes

Reviewer #4: Yes

**********

Reviewer #3: Author has responded very well to the earlier comments given by reviewers and has addressed all the required changes. He also applied recommendations given, even though he justified why it wasn't not in the initial manuscript.

He used real data to support his findings abs did a rigorous analysis.

I suggest approval for this manuscript.

Reviewer #4: Integrating Decision Trees with ANOVA for Enhanced Interaction Visualisations

The authors present a regression tree based web-app to succinctly visualize

interactions from ANOVA hierarchically. I appreciate the authors' goal to

simplify understanding and communicating high-order interaction terms, but it is

unclear to me if the described approach accomplishes that goal. I have some

questions about the approach, as well as a couple points of disagreement.

- You wrote repeatedly that your approach shows the "hierarchical structure" of

the interaction. Interactions in ANOVAs of the sort you discuss have no

hierarchy. They are simultaneous functions of all involved variables. I

believe you know this and that you mean something else, like a ranking of sums

of squares or ranking of binary-split mean differences. I believe you

mentioned a few times that readers and researchers struggle with

interactions. If you tell them several times that their interactions have

hierarchical structure, will they not come away from your work thinking that?

That won't be helpful. Helping people understand interactions is

important. Trying to accomplish that by turning them into something they are

not, like hierarchical trees, is unhelpful. In some sense, it parallels some

methodologists' recommendations to look at simple slopes and now many applied

researchers turn their interactions into a simple slopes plot and just

interpret simple slopes. That is inadequate too.

- You also wrote at least a couple times that your EXCITE approach removes the

need for post-hoc means testing. At least that is how I understood you. I

believe you asserted this, but I don't think you explained it. I also don't

think it could be true, nor does it seem reasonable on logical grounds.

For example, if I looked at my data in a way similar to your tree in Figure

10, meaning I started with some marginal difference that I expected (or knew)

was biggest and then looked at mean differences by factor 2 within levels of

factor 1, I'd have the middle of your tree. Then if I just looked at all eight

cell means, I'd have the bottom of your figure. If I do this by hand in R or

SPSS, write it up and not at least discuss p-value correction, I'd hope and

expect reviewers to be highly critical. You seem to be saying that if a

regression tree does all those comparisons for you and just shows you the

ones it arrived at, that is different. Why?

On lines 604 and 605, you write that EXCITE highlights the influential

combinations directly, but the algorithm is checking all the combinations too,

isn't it?

- You also treat the p-value like a very concrete entity, linking your tuning

parameter to it. Aren't p-values more heuristic and generally informative,

rather than a hard and fast measure of support. Methods researchers have been

encouraging people to rely less on p-values and more on effect sizes for

decades. You appear to be putting a lot on the p-value.

Relatedly, we often have quite low power for interactions. Rather than tuning

low power and likely poorly estimated terms away, with more aggressive

pruning, shouldn't we be encouraging researchers to think more clearly about

high-order interactions, and ways to test and understand them better?

- lines 208 to 217, I think you are saying that your method identifies important

factors even in the presence of multicollinearity. My understanding of the

issue is that we cannot usually identify the problem variable in a

multicollinear regression problem, which of course includes your ANOVA

problems. Taking your example even, "teaching method" and "school type" may be

highly associated and therefore your tree algorithm attributes the variance to

teaching method and omits school type from the final model. That presents the

results much more concretely than is warranted. An algorithm picks teaching

method, but if they are collinear, we don't actually know what's most

important. If I understand this section, I think you are saying the researcher

would be told one factor is the important one, when in reality, we don't

really know.

- I don't think you really explain, nor illustrate, why we want the

"cost-complexity pruning" you introduce on line 254. If I have done a good

experiment and been told I need some k-order interaction to understand the

pattern of means, why isn't that the right place to start? Why do I want a

tree algorithm to tell me to ignore smaller effects? I'm doing science after

all, not trying to have simple results or small tables.

- lines 530 and 531: is it bad that we ask scientists and readers to take time

to think through and work to understand interactions? They can be

complicated. Complicated things can take time. That's okay, isn't it?

- Last, is your three-way ANOVA example good? All the partial eta^2 CIs are

huge, like all (or nearly all) the possible numeric range. When I see

something like that, I usually thinks there is something wrong with the

data. Is such a weak data example good for this paper? If you look for a new

example, maybe include one that illustrates more of the benefit of bringing

these trees to your purpose.

- In your response to Reviewer 1, you give a rationale for only discussing 2-

and 3-way ANOVA interactions. But on line 146, you introduce n-way AVOVA. That

seems unnecessary and adds rather little to the content. For example, you

don't discuss higher-order terms. Notationally, it is also confusing. Earlier,

you used i,j,k, but here it is i_1, i_2, ..., and then r.

- on line 157, you point out that researchers frequently omit higher-order

interactions... "to simplify interpretation". Can you add the words "lazily

and sloppily", as in "...researchers frequently lazily and sloppily omit

higher-order interactions..." That is only half a joke. Can you convey that

while people frequently do omit higher-order terms to make their work easier,

that that is not how one does good science?

typos, corrections, etc.

- line 135, where should be lower case and I assume a comma should follow the

equation

- line 186, you write "optimal", but optimal what?

- line 221, rpart is implemented in a package or library, not in a manual.

- figure 10, I think one of the Cyberballs should be Uberball

**********

what does this mean?). If published, this will include your full peer review and any attached files.

If you choose “no”, your identity will remain anonymous but your review may still be made public.

**Do you want your identity to be public for this peer review?**  For information about this choice, including consent withdrawal, please see our Privacy Policy

Reviewer #3: **Yes:** Samuel Sarpong Fosu

Reviewer #4: No

**********

[NOTE: If reviewer comments were submitted as an attachment file, they will be attached to this email and accessible via the submission site. Please log into your account, locate the manuscript record, and check for the action link "View Attachments". If this link does not appear, there are no attachment files.]

To ensure your figures meet our technical requirements, please review our figure guidelines: https://journals.plos.org/plosone/s/figures

You may also use PLOS’s free figure tool, NAAS, to help you prepare publication quality figures: https://journals.plos.org/plosone/s/figures#loc-tools-for-figure-preparation.

NAAS will assess whether your figures meet our technical requirements by comparing each figure against our figure specifications.

# Author comment: Author response to Decision Letter 2

**DOI:** 10.1371/journal.pone.0357663.r004

31 Mar 2026

Iftikhar Ahmed Khan, Academic Editor

PLOS ONE

March 25, 2026

Dear Editor and Reviewer(s),

Thank you for taking the time to review our work (PONE-D-25-38509R1) and providing us with insightful feedback on ways to strengthen our paper. We have incorporated the suggested changes and addressed each point below.

We believe that the revised version better communicates the novelty and significance of our work. We are confident that our web application stands as a valuable addition to the existing body of knowledge in this field.

We hope that the revised version meets your expectations.

Thank you for your time and consideration.

On behalf of all the authors,

Yours sincerely,

Perseverence Savieri

Corresponding Author

Biostatistics and Medical Informatics Research Group (BISI)

Vrije Universiteit Brussel (VUB), Brussels 1090, Belgium

Perseverence.Savieri@vub.ac.be

perseverencesavieri@gmail.com

Manuscript ID: PONE-D-25-38509R1

Manuscript title: Integrating Decision Trees with ANOVA for Enhanced Interaction Visualisations

Response to Reviewer #4:

We thank the reviewer for the thoughtful and methodologically engaged comments. You raise several conceptual points regarding the interpretation of interactions and the positioning of the EXCITE framework. We have revised the manuscript to clarify these aspects. Importantly, EXCITE is designed as a visualisation and interpretation tool that complements classical ANOVA, rather than replacing its inferential framework. Below, we address each comment in detail.

Major comments

Comment 1: Interactions do not have a hierarchical structure

We agree with the reviewer that ANOVA interaction terms are not hierarchical. Interactions represent simultaneous conditional relationships among factors, and our intention was not to imply otherwise. Instead, the hierarchy arises from the visual representation imposed by recursive partitioning, which orders conditional mean differences according to their contribution to variance reduction. In other words, the hierarchy in the trees reflects the structure of the tree visualisation, not the statistical structure of the interaction itself. You are right that we should make this very clear.

Nonetheless, tree-based representations of interactions are widely used in statistical learning because they provide an interpretable representation of conditional relationships (Breiman et al., 1984; Hastie et al., 2009). In this context, our trees should be interpreted as a visual ordering of conditional effects, analogous to the way interaction plots organise effects across panels. To avoid possible misunderstanding, we clarified this explicitly in the manuscript (lines 174-176) and revised several occurrences of the term “hierarchical” so that it refers only to the structure of the tree representation rather than to the interaction itself.

Comment 2: The method appears to remove the need for post-hoc testing.

We appreciate the opportunity to clarify this point. EXCITE does not replace formal post-hoc inference, and this was not our intention. Rather, EXCITE addresses a common practical issue: researchers often conduct many exploratory pairwise comparisons to identify interaction patterns, and these comparisons can proliferate rapidly as the number of factor levels increases. The decision tree provides a data-driven visual decomposition of conditional means, allowing researchers to identify influential factor combinations before conducting confirmatory contrasts. However, this exploratory step does not eliminate the need for formal statistical inference. When specific contrasts are tested, appropriate adjustments for multiple comparisons (e.g., Bonferroni, Tukey, or similar procedures) remain necessary to control Type I error. To avoid ambiguity, we have revised the manuscript to state that EXCITE complements explicitly, but does not replace, post-hoc inference, and that multiplicity correction remains essential when evaluating statistical significance (lines 609–612).

Comment 3: You write that EXCITE highlights the influential combinations directly, but the algorithm is checking all the combinations too, isn't it?

We appreciate this clarification request. The reviewer is correct that the CART algorithm evaluates many possible candidate splits internally when constructing the regression tree. In that sense, the algorithm implicitly considers multiple possible partitions of the factor levels. However, the purpose of the decision-tree representation in EXCITE is not to enumerate all possible comparisons but to identify and display the partitions that produce the largest reductions in within-node variance, as defined by the CART splitting criterion (Breiman et al., 1984). In other words, although many candidate combinations are evaluated during tree construction, the final visualisation presents only the subset of conditional splits that most strongly structure the response variable. This behaviour differs from manually examining all cell means or conducting numerous pairwise comparisons, where every contrast must be inspected separately and, importantly, requires appropriate correction for multiple testing. The decision tree, therefore, provides a structured summary of the most influential conditional relationships, which can guide subsequent confirmatory analyses. When formal comparisons are conducted based on these patterns, standard multiplicity adjustments remain necessary to ensure valid statistical inference. To clarify this point, we added a short explanation in the manuscript (lines 183-187 and lines 618-631).

Comment 4: The method relies heavily on p-values when tuning the complexity parameter.

The reviewer raises an important point regarding the interpretation of p-values. In EXCITE, the p-value is used only as a heuristic to guide tree complexity, not as a strict inferential criterion. Specifically, the mapping between interaction significance and the complexity parameter aligns the depth of the tree visualisation with the strength of the statistical evidence. This approach prevents overly complex trees when interaction evidence is weak, while still allowing deeper partitions when interactions are strongly supported. Importantly, the ANOVA output displayed in the application includes effect sizes (partial η²) alongside p-values, and the interpretation of results should always consider both. We have clarified this point in the manuscript (lines 286-289). With respect to the reviewer’s concern about the typically low statistical power of interaction effects, it is important to note that the pruning rule affects only the visual tree representation and does not alter the underlying factorial ANOVA results. All estimated interaction terms remain available in the statistical output, allowing researchers to investigate higher-order effects directly, even when the tree representation is simplified for interpretability. The pruning rule therefore serves to stabilise the visualisation and reduce over-interpretation of small or unstable subgroup splits, rather than discouraging careful examination of interaction effects. We agree that alternative criteria, such as effect-size–based thresholds, could provide a valuable complement to p-value–guided pruning. The current implementation represents a first, pragmatic approach aligned with conventional ANOVA outputs, but future versions of EXCITE could incorporate effect-size–driven or hybrid criteria to guide tree complexity. We have added this as a limitation and direction for future development in the manuscript (lines 695-697).

Comment 5: The multicollinearity example may imply that the algorithm identifies the most influential variable.

We agree with the reviewer that when predictors are strongly correlated, recursive partitioning methods, like regression models, cannot reliably distinguish which variable is most influential. In such cases, the allocation of variance to a particular predictor depends on the structure of the data and the splitting criterion, and different but highly correlated predictors may lead to similar partitions. The purpose of the example was to illustrate that variable importance measures can reflect how predictors contribute to variance partitioning within the tree, even if they are not selected as splitting variables in the final pruned tree. However, we acknowledge that in the presence of multicollinearity, these measures should not be interpreted as uniquely identifying the most important predictor. We have revised the manuscript to clarify that variable importance reflects the role of predictors within the tree representation and should be interpreted with caution when predictors are correlated (lines 309–315).

Comment 6: Why use cost-complexity pruning if higher-order interactions are scientifically meaningful?

Cost-complexity pruning is a standard component of CART methodology (Breiman et al., 1984) and is used to prevent overfitting caused by recursive splitting of small subsets. The goal in EXCITE is not to suppress meaningful interactions but to ensure that the tree visualisation remains stable and interpretable. Without pruning, regression trees often grow deep structures that reflect sampling noise rather than meaningful patterns. Importantly, the ANOVA results remain fully available in the interface, meaning that all estimated interaction terms remain accessible even when the visual tree is pruned (lines 261-265).

Comment 7: Is it problematic that interactions require time to interpret?

We agree that complex interactions often require careful interpretation. The aim of EXCITE is not to oversimplify statistical reasoning but to provide a visual framework that helps researchers organise conditional relationships more efficiently. In many applied settings, interaction plots across multiple panels can be difficult to synthesise visually. The tree representation provides a complementary view that consolidates these conditional relationships in a single diagram.

Comment 8: Concern about the three-way ANOVA example and wide effect-size intervals.

We thank the reviewer for this important observation. We agree that the wide confidence intervals for the partial η² estimates indicate that the effect sizes are estimated with considerable uncertainty, making it difficult to distinguish precisely between small and larger effects based on these estimates alone. This imprecision likely reflects the limited information available for estimating higher-order interaction effects in the dataset, a common issue in factorial designs where interaction effects tend to have lower statistical power and greater variability than main effects.

The purpose of this example is not to emphasise precise effect-size estimation, but to illustrate how EXCITE visualises conditional interaction structures in a realistic applied setting. Importantly, the interaction pattern itself is consistent with the findings reported in the original study (Thériault et al., 2023), and the decision tree representation focuses on the relative structure of subgroup differences rather than the magnitude of effect sizes. To address the reviewer’s concern, we have revised the manuscript to explicitly acknowledge the uncertainty in the effect-size estimates and clarify that the example is intended for visualisation purposes rather than precise effect-size interpretation (lines 507-510).

Comment 9: n-way ANOVA notation is unnecessary and adds rather little to the content.

We agree and have removed the general n-way notation to improve clarity.

Comment 10: Suggestion regarding wording on higher-order interactions

While we appreciate the reviewer’s humour, we felt that adopting this wording might introduce an unintended tone. However, we agree with the underlying point that higher-order interactions should not be omitted purely for convenience. To reflect this, we have revised the manuscript to emphasise that while higher-order interactions are often excluded to simplify interpretation or preserve statistical power, model specification should be guided by theoretical considerations and study design rather than convenience alone. This clarification has been added in the Methods section (lines 148–150).

Minor corrections

• All typographical issues identified by the reviewer were corrected.

• Figure 10 clarification: The reviewer suggested a possible labelling error in Figure 10. We carefully checked the figure and confirmed that the labels are correct. No changes were required.

We thank the reviewer again for the constructive comments, which helped improve the clarity and conceptual positioning of the manuscript

Attachment Submitted filename: Response_to_Reviewers_auresp_2.docx

# Peer review: Decision Letter 2

**DOI:** 10.1371/journal.pone.0357663.r005

**Authors:** Iftikhar Khan

6 Jul 2026

Dear Dr. Savieri,

Thank you for submitting your manuscript to PLOS ONE. After careful consideration, we feel that it has merit but does not fully meet PLOS ONE’s publication criteria as it currently stands. Therefore, we invite you to submit a revised version of the manuscript that addresses the points raised during the review process. The reviewer though recommended acceptance, yet raise few questions for calrification. You are requested to address the query and resubmit.

Please submit your revised manuscript by Aug 20 2026 11:59PM. If you will need more time than this to complete your revisions, please reply to this message or contact the journal office at plosone@plos.org. When you're ready to submit your revision, log on to https://www.editorialmanager.com/pone/ and select the 'Submissions Needing Revision' folder to locate your manuscript file.

- A letter that responds to each point raised by the academic editor and reviewer(s). You should upload this letter as a separate file labeled 'Response to Reviewers'.
- A marked-up copy of your manuscript that highlights changes made to the original version. You should upload this as a separate file labeled 'Revised Manuscript with Track Changes'.
- An unmarked version of your revised paper without tracked changes. You should upload this as a separate file labeled 'Manuscript'.

If you would like to make changes to your financial disclosure, please include your updated statement in your cover letter. Guidelines for resubmitting your figure files are available below the reviewer comments at the end of this letter.

If applicable, we recommend that you deposit your laboratory protocols in protocols.io to enhance the reproducibility of your results. Protocols.io assigns your protocol its own identifier (DOI) so that it can be cited independently in the future. For instructions see: https://journals.plos.org/plosone/s/submission-guidelines#loc-laboratory-protocols. Additionally, PLOS ONE offers an option for publishing peer-reviewed Lab Protocol articles, which describe protocols hosted on protocols.io. Read more information on sharing protocols at https://plos.org/protocols?utm_medium=editorial-email&utm_source=authorletters&utm_campaign=protocols.

As the corresponding author, your ORCID iD is verified in the submission system and will appear in the published article. PLOS supports the use of ORCID, and we encourage all coauthors to register for an ORCID iD and use it as well. Please encourage your coauthors to verify their ORCID iD within the submission system before final acceptance, as unverified ORCID iDs will not appear in the published article. *Only*  the individual author can complete the verification step; PLOS staff *cannot*  verify ORCID iDs on behalf of authors.

We look forward to receiving your revised manuscript.

Kind regards,

Iftikhar Ahmed Khan

Academic Editor

PLOS One

Journal Requirements:

1. If the reviewer comments include a recommendation to cite specific previously published works, please review and evaluate these publications to determine whether they are relevant and should be cited. There is no requirement to cite these works unless the editor has indicated otherwise.

2. Please review your reference list to ensure that it is complete and correct. If you have cited papers that have been retracted, please include the rationale for doing so in the manuscript text, or remove these references and replace them with relevant current references. Any changes to the reference list should be mentioned in the rebuttal letter that accompanies your revised manuscript. If you need to cite a retracted article, indicate the article’s retracted status in the References list and also include a citation and full reference for the retraction notice.

[Note: HTML markup is below. Please do not edit.]

Reviewers' comments:

Reviewer's Responses to Questions

**Comments to the Author**

Reviewer #4: (No Response)

**********

2. Is the manuscript technically sound, and do the data support the conclusions?

Reviewer #4: Yes

**********

3. Has the statistical analysis been performed appropriately and rigorously?

Reviewer #4: Yes

**********

4. Have the authors made all data underlying the findings in their manuscript fully available?

The PLOS Data policy

Reviewer #4: Yes

**********

5. Is the manuscript presented in an intelligible fashion and written in standard English?

Reviewer #4: Yes

**********

Reviewer #4: Integrating Decision Trees with ANOVA for Enhanced Interaction Visualisations

PONE-D-25-38509R2

Dear authors, Thank you for answering my questions and explaining your

perspective and thinking. I understand your goals and procedure better.

Upon reading the second revision, I had a few more questions or points of

clarification.

- lines 269--271: You write that "... p_{int} denotes the smallest omnibus

interaction p-value..., representing the strength of the evidence for the

highest order interaction." While it may be common that the highest order

interaction often has the largest p-value, that isn't a mathematical

certainty, is it? Couldn't a mean square for a two-way interaction, in a

three factor experiment have a smaller p-value than the three-way interaction?

If this is correct, that it is possible, then shouldn't p_{int} always be the

p-value for the highest order interaction, "representing the strength of the

evidence for the highest order interaction"?

- line 285, the difference between "all possible interactions" and "model

interactions" isn't clear to me in the context of your paper. It sounds like

EXCITE starts with the three-way interaction, which would include all possible

interactions. Then what can "model interactions" be? It sounds like it would

be for a user specified model that did not include all possible interactions,

but you don't really discuss, nor does it sound like EXCITE does this.

- line 466, I know you added language specifying that interactions are not

hierarchical, which I appreciate. However, on line 466, you describe

interaction effects "unfold"ing. Maybe I'm being too fussy, but this suggests

a process in my mind, which again, I think is going to confuse matters for

your potential users. Your readers are people who want help with two- and

three-way interactions in ANOVA, which are among the easiest interactions to

interpret. These readers do not need language that creates a false

impression. ANOVA interactions don't unfold. As you write, they are

simultaneous conditional associations. I think it would be preferrable to say

something like ... showing how the main and interaction effects are

characterized by successive splits.

- Last, in a few places you describe how CART can misleadingly attribute

variance, and therefore importance, to one of a set of collinear

variables. This seems like a difficulty easily solved in software. Again, I

presume your users are people who aren't very statistically savvy. They may

not have a good understanding of multicollinearity. Despite your statements

about possible collinearity and CART not flagging that, many users are likely

to take the CART results as correct. Can you add something to EXCITE to check

multicollinearity, perhaps separate from the cart procedure, and issue a

noticeable warning or otherwise make it difficult for the user to ignore?

**********

what does this mean?). If published, this will include your full peer review and any attached files.

If you choose “no”, your identity will remain anonymous but your review may still be made public.

**Do you want your identity to be public for this peer review?**  For information about this choice, including consent withdrawal, please see our Privacy Policy

Reviewer #4: No

**********

[NOTE: If reviewer comments were submitted as an attachment file, they will be attached to this email and accessible via the submission site. Please log into your account, locate the manuscript record, and check for the action link "View Attachments". If this link does not appear, there are no attachment files.]

To ensure your figures meet our technical requirements, please review our figure guidelines: https://journals.plos.org/plosone/s/figures

You may also use PLOS’s free figure tool, NAAS, to help you prepare publication quality figures: https://journals.plos.org/plosone/s/figures#loc-tools-for-figure-preparation.

NAAS will assess whether your figures meet our technical requirements by comparing each figure against our figure specifications.

# Author comment: Author response to Decision Letter 3

**DOI:** 10.1371/journal.pone.0357663.r006

12 Aug 2026

Manuscript ID: PONE-D-25-38509R2

Manuscript title: Integrating Decision Trees with ANOVA for Enhanced Interaction Visualisations

Reviewer #4:

Weaknesses highlighted

Comment 1: Clarification of the definition of p_int

We thank the reviewer for identifying this ambiguity. The implementation of EXCITE does not use the smallest interaction p-value across all interaction terms. Rather, it uses the p-value corresponding to the highest-order interaction term (i.e., the two-way interaction in a two-factor ANOVA or the three-way interaction in a three-factor ANOVA) to determine the complexity parameter. We have therefore revised the manuscript (lines 269-272) to remove the phrase "smallest omnibus interaction p-value" and instead explicitly state that p_int denotes the omnibus p-value for the highest-order interaction. This wording more accurately reflects both the methodology and software implementation.

Comment 2: Clarification of tree complexity options

Thank you for this helpful observation. We agree that the previous label "Model interactions" was ambiguous and did not accurately reflect the functionality of EXCITE. The application always fits the complete factorial ANOVA model; the available options control only how the resulting decision tree is displayed and pruned, rather than which interaction terms are included in the ANOVA model. To address this, we simplified the user interface by replacing the previous interaction labels with two tree display modes: Maximum tree complexity and ANOVA-guided tree (recommended). We also revised the corresponding manuscript text (lines 284-304 and 390-394) to clarify that these options control the degree of pruning applied to the decision tree while leaving the underlying ANOVA model unchanged.

Comment 3: Wording describing interaction effects

We thank the reviewer for this helpful suggestion. We agree that the term "unfold" could inadvertently imply a sequential process rather than the simultaneous conditional relationships represented by ANOVA interactions. We have revised the wording accordingly (lines 476-477) to clarify that the successive tree splits provide a hierarchical representation of the conditional structure of the fitted ANOVA model rather than suggesting that interaction effects develop sequentially.

Comment 4: Handling multicollinearity in EXCITE

We appreciate the reviewer's suggestion. We agree that multicollinearity can influence CART variable-importance measures and may not be readily recognised by inexperienced users. However, EXCITE was intentionally designed as an interaction interpretation and visualisation tool that operates after a factorial ANOVA model has been specified, rather than as a comprehensive model-building or diagnostic platform. Incorporating multicollinearity diagnostics would substantially broaden the scope of the application beyond its intended purpose and would logically require the inclusion of other model diagnostics, such as checks for normality, homoscedasticity, independence, and influential observations. Instead, we have strengthened the discussion of this limitation in the manuscript (lines 673-680) by explicitly advising users to assess multicollinearity and other model assumptions prior to interpreting the decision tree and its variable-importance measures.

Attachment Submitted filename: Response_to_Reviewers_auresp_3.docx

# Editor report: Decision Letter 3

**DOI:** 10.1371/journal.pone.0357663.r007

**Authors:** Iftikhar Khan

20 Aug 2026

Integrating Decision Trees with ANOVA for Enhanced Interaction Visualisations

PONE-D-25-38509R3

Dear Dr. Savieri,

We’re pleased to inform you that your manuscript has been judged scientifically suitable for publication and will be formally accepted for publication once it meets all outstanding technical requirements.

Within one week, you’ll receive an e-mail detailing the required amendments. When these have been addressed, you’ll receive a formal acceptance letter and your manuscript will be scheduled for publication.

An invoice will be generated when your article is formally accepted. Please note, if your institution has a publishing partnership with PLOS and your article meets the relevant criteria, all or part of your publication costs will be covered. Please make sure your user information is up-to-date by logging into Editorial Manager at Editorial Manager® and clicking the ‘Update My Information' link at the top of the page. For questions related to billing, please contact billing support.

If your institution or institutions have a press office, please notify them about your upcoming paper to help maximize its impact. If they’ll be preparing press materials, please inform our press team as soon as possible -- no later than 48 hours after receiving the formal acceptance. Your manuscript will remain under strict press embargo until 2 pm Eastern Time on the date of publication. For more information, please contact onepress@plos.org.

Kind regards,

Iftikhar Ahmed Khan

Academic Editor

PLOS One

Additional Editor Comments (optional):

Reviewers' comments:

# Editor report: Acceptance letter

**DOI:** 10.1371/journal.pone.0357663.r008

**Authors:** Iftikhar Khan

PONE-D-25-38509R3

PLOS One

Dear Dr. Savieri,

I'm pleased to inform you that your manuscript has been deemed suitable for publication in PLOS One. Congratulations! Your manuscript is now being handed over to our production team.

At this stage, our production department will prepare your paper for publication. This includes ensuring the following:

* All references, tables, and figures are properly cited

* All relevant supporting information is included in the manuscript submission,

* There are no issues that prevent the paper from being properly typeset

You will receive further instructions from the production team, including instructions on how to review your proof when it is ready. Please keep in mind that we are working through a large volume of accepted articles, so please give us a few days to review your paper and let you know the next and final steps.

Lastly, if your institution or institutions have a press office, please let them know about your upcoming paper now to help maximize its impact. If they'll be preparing press materials, please inform our press team within the next 48 hours. Your manuscript will remain under strict press embargo until 2 pm Eastern Time on the date of publication. For more information, please contact onepress@plos.org.

You will receive an invoice from PLOS for your publication fee after your manuscript has reached the completed accept phase. If you receive an email requesting payment before acceptance or for any other service, this may be a phishing scheme. Learn how to identify phishing emails and protect your accounts at https://explore.plos.org/phishing.

If we can help with anything else, please email us at customercare@plos.org.

Thank you for submitting your work to PLOS One and supporting open access.

Kind regards,

PLOS One Editorial Office Staff

on behalf of

Dr. Iftikhar Ahmed Khan

Academic Editor

PLOS One
